# Time-course transcriptomic information unravels the adaptation strategies of *Nicotiana tabacum* to drought stress through altered root system architecture

**DOI:** 10.3389/fpls.2026.1781718

**Published:** 2026-04-06

**Authors:** Yu Zhang, Yi Wang, Hai Hu, Hui Wang, Chenggang Luo

**Affiliations:** 1 Tobacco Research Institute of Chinese Academy of Agricultural Sciences China, Qing’dao, China; 2 Weifang Tobacco Co., Ltd., Weifang, China; 3 China Tobacco Hebei Industrial Co., Ltd, Shijiazhuang, China; 4 Qingdao Institute of Bioenergy and Bioprocess Technology Academy of Sciences, Shangdong Energy Institute, Qing’dao, China

**Keywords:** drought resilience, root traits, tobacco, transcriptomics, water scarcity

## Abstract

**Introduction:**

Drought stress, exacerbated by global climate change, poses a significant threat to agricultural productivity and global food security. Plants have evolved intricate adaptive mechanisms, with the root system playing a crucial role in sensing soil water deficits and acquiring essential resources.

**Methods:**

This study investigated the time-course adaptation strategies of two *Nicotiana tabacum* varieties, ZC208 (drought-tolerant) and ZY100 (drought-sensitive), to different levels of drought stress through altered root system architecture (RSA) and transcriptomic regulations.

**Results:**

RSA parameters generally decreased, while specific root length, surface area, and volume increased. Antioxidant enzyme activities (SOD, POD, CAT) and proline content in roots significantly increased, contrasting with a reduction in soluble sugars. This research provides valuable insights into the dynamic molecular and physiological mechanisms underlying drought adaptation in tobacco roots, offering potential targets for breeding drought-tolerant cultivars to enhance agricultural resilience in a changing climate. Analysis of the root transcriptome identified a large cohort of differentially expressed genes (DEGs), with more downregulated than upregulated DEGs across all drought stages in both varieties. Time-course analysis using STEM identified distinct expression profiles for ZC208 (6 profiles) and ZY100 (8 profiles), highlighting dynamic gene regulation over time. WGCNA identified modules strongly correlated with RSA parameters, with key pathways including ‘MAPK signaling pathway, plant’, ‘plant-pathogen interaction’, and ‘sesquiterpenoid and triterpenoid biosynthesis’. Genes involved in plant hormone signaling (e.g., AUX/IAA, PYR/PYL) and starch/sucrose metabolism (e.g., endoglucanase, β-glucosidase) showed differential expression patterns, indicating their crucial roles in drought response.

**Discussion:**

This research provides valuable insights into the dynamic molecular and physiological mechanisms underlying drought adaptation in tobacco roots, offering potential targets for breeding drought-tolerant cultivars to enhance agricultural resilience in a changing climate.

## 1 Introduction

Global climate change has led to a significant increase in the frequency and intensity of drought stress, posing a severe threat to global agricultural production (Alotaibi, 2023). Drought is recognized as one of the most destructive abiotic stressors, severely inhibiting crop growth, reducing yields, and jeopardizing food security worldwide (Vadez et al., 2024). Projections indicate that by 2050, over 50% of the world’s agricultural land will be severely damaged by drought, adversely affecting plant productivity and global food security (Hong et al., 2015; Kapoor et al., 2020; Spinoni et al., 2018). Insufficient precipitation, elevated temperatures, and human activities such as intense cultivation and deforestation further diminish soils’ ability to store and assimilate water, exacerbating the impacts of drought (Raposo et al., 2023). This escalating water scarcity not only leads to reduced crop yields and soil degradation but also hinders plant growth (Anderson et al., 2020; Babić et al., 2024), thereby threatening the stability of global food systems (Bogati and Walczak, 2022). Therefore, an in-depth understanding of plant responses to drought stress is crucial for developing resilient agricultural practices and ensuring future food security (Bogati and Walczak, 2022).

Plants have evolved a complex array of adaptive mechanisms at morphological, physiological, biochemical, and molecular levels to cope with drought stress (Hammond et al., 2020; Seleiman et al., 2021). Among plant organs, roots are pivotal in sensing soil water deficits and acquiring water and nutrients, making them the first line of defense against drought (Seleiman et al., 2021; Siddiqui et al., 2021). Under drought conditions, plants modify their root system architecture (RSA) by developing deeper roots, increasing root angles, enhancing root hair density, and thickening root xylem to more efficiently access water from deeper soil layers (Castro et al., 2019; Gupta et al., 2021). These architectural changes are integral to maintaining plant productivity during drought (Williams and de Vries, 2020). At the physiological and biochemical levels, roots accumulate osmolytes such as proline and soluble sugars to maintain cell turgor and membrane stability, and to scavenge reactive oxygen species (ROS) (Chieb and Gachomo, 2023; Schieber and Chandel, 2014). Drought also induces the activation of antioxidant enzyme systems, including superoxide dismutase (SOD), catalase (CAT), peroxidase (POD), and ascorbate peroxidase (APX), in roots to mitigate oxidative damage caused by excessive ROS accumulation (Ren et al., 2024). At the molecular level, transcription factors (TFs) regulate the expression of numerous genes involved in hormone signaling—such as abscisic acid (ABA), ethylene (ET), jasmonic acid (JA), and salicylic acid (SA)—and the biosynthesis of secondary metabolites (e.g., flavonoids, lignin), which strengthen cell walls and reduce water loss (Lei et al., 2023; Shao et al., 2023; Zhi et al., 2024). Transcriptomic analysis has emerged as a powerful tool for unraveling the gene networks and regulatory pathways underlying plant root responses to water deficit conditions (Lake et al., 2017; Nakashima et al., 2014). For instance, studies on *Seriphidium transiliense* revealed that more genes in roots were affected by drought stress than in leaves (Liu et al., 2022), while transcriptome analysis in Leucaena plants identified significant physiological changes and gene expression adaptations in both leaves and roots contributing to its resilience (Zhi et al., 2024). Similarly, multi-omics joint analysis in soybean roots clarified differences in gene expression and drought-resistance pathways, identifying phenylpropanoid and isoflavonoid biosynthesis as core drought resistance pathways (Wang et al., 2024).

This study aims to comprehensively investigate the effects of different levels of drought stress on the root system architecture and conduct time-course transcriptomic analysis in two tobacco varieties: Zhongchuan 208 (ZC208, drought-tolerant) and Zhongyan 100 (ZY100, drought-sensitive). By comparing the changes in root morphology, physiological and biochemical responses, and gene expression patterns between the two varieties under drought stress, this research seeks to deepen our understanding of the adaptive mechanisms of tobacco roots to drought. The time-course transcriptomic information will unravel the dynamic molecular regulations underlying these adaptation strategies. The findings will provide theoretical insights and molecular targets for screening and breeding drought-tolerant tobacco varieties, thereby contributing to addressing the agricultural challenges posed by global climate change.

## 2 Materials and methods

### 2.1 Plant materials and experiment design

Seeds of two *Nicotiana tabacum* (*N. tabacum*) varieties, ZC208(drought-tolerant) and ZY100(drought-sensitive), were used in this study. ZC208 and ZY100 are two widely cultivated tobacco varieties in China. Based on our preliminary field observations, ZC208 exhibits consistent phenotypic tolerance to water-limited conditions, whereas ZY100 is known to be more sensitive to drought stress. Therefore, selecting these two contrasting varieties provides an ideal system for comparative analysis to uncover the genetic and physiological basis of drought tolerance in *N. tabacum*. Seeds were germinated and grown in a controlled environment chamber under optimal conditions (25 °C day/night, 14 h light/10 h dark cycle, 67-70% humidity, 600 µmol m^-^² s^-^¹ photosynthetically active radiation). After the third true leaf expanded, healthy seedlings were transplanted individually into plastic pots containing 2.0 kg of air-dried soil (**Supplementary Table S1**). Compound fertilizer (N-P-K, 15-15–15 ratio) was applied as basal fertilization, and deionized water was used for daily irrigation to avoid nutrient deficiency and water shortages.

Random assignment was used to divide the two-week-old, uniformly grown plants into two groups prior to initiating the water treatments: (1) Control (CK), maintained at a soil water content of 73–75% of field capacity (FC); (2) Drought (D), maintained at a soil water content of 38–40% of FC. Soil water content was measured and adjusted every 7 days by artificial watering. Root samples from both CK and D treatments were collected at five time points: 0, 14, 21, 28, and 35 days after the initiation of water treatments. The five stages of drought stress were designated as DS7, DS14, DS21, DS28, and DS35, respectively. For each sampling time point and treatment, six biological replicates were collected: three were used for the analysis of root biomass and structural indicators, while the remaining three were harvested for physiological and transcriptomic analyses. To minimize environmental interference and maintain the stress-induced physiological state, the latter samples were subjected to a rapid ‘rinse-and-freeze’ protocol. Upon removal from the pots, roots were gently shaken to remove bulk soil, rinsed with ice-cold deionized water for no more than 10 seconds to remove adhering fine particles, blotted dry with sterile filter paper, and immediately flash-frozen in liquid nitrogen for storage at -80 °C. This approach was adopted to prevent RNA degradation and the leaching of soluble osmolytes, as well as to avoid the activation of wound- or water-immersion-related signaling pathways.

### 2.2 Root ultrastructure observation

Ultrastructural analysis of root samples was performed via transmission electron microscopy (TEM). The preparation protocol involved the fixation of small root segments (~1–2 mm) in 2.5% glutaraldehyde within a 0.1 M phosphate buffer (pH 7.4) for 24 h at 4 °C. Post-fixation was carried out with 1% osmium tetroxide for 2 h at room temperature after a rinsing step. Dehydration was then achieved using a graded ethanol series (30%-100%), followed by embedding in Spurr’s resin. An ultramicrotome (Leica EM UC7, Germany) was used to obtain ultrathin sections (70–90 nm). These sections underwent double staining with uranyl acetate and lead citrate before observation with a transmission electron microscope (Hitachi H-7650, Japan).

### 2.3 Root biomass and structural indicators

Root samples were carefully washed to remove soil particles. For root biomass, fresh weight (FW) was measured immediately, and then samples were oven-dried at 80 °C for 48 hours to determine dry weight (DW). Root structural indicators, including total root length (RL), root surface area (RSA), root volume (RV), projected area (PA), average diameter (AD), length per volume (LPV), specific root length (SRL), specific root surface area (SRSA), specific root volume (SRV), number of tips (TIP), forks (FOR), and crossings (CRO), were evaluated using a root scanner (Epson Expression 12000XL, Japan) and analyzed with WinRHIZO software (Regent Instruments Inc., Canada) (Tiwari et al., 2021). The phenotypic images obtained from root scanning are illustrated in **Supplementary Figure 1**.

### 2.4 Root physiological indices

Superoxide anion (O_2_^•^-^^) content was measured using the hydroxylamine oxidation method (Li et al., 2017). Hydrogen peroxide (H_2_O_2_) content was determined spectrophotometrically after reaction with potassium iodide (Liu et al., 2020). Hydroxyl radical (^•^OH) content was assessed using the salicylic acid method (Saleem et al., 2024). Malondialdehyde (MDA) content, an indicator of lipid peroxidation, was measured using the thiobarbituric acid (TBA) method (Madhava Rao and Sresty, 2000). Root activity (RA) was determined by the triphenyltetrazolium chloride (TTC) reduction method (Salehi et al., 2016). For enzyme activities, the nitrogen blue tetrazole (NBT) photochemical reduction method (Cvetkovic et al., 2017) was employed for superoxide dismutase (SOD), the guaiacol method (Zhang and Kirkham, 1994) for peroxidase (POD), and the ammonium molybdate method (Pomeroy et al., 1975) for catalase (CAT). For osmolyte contents, the anthrone-sulfuric acid method (Kohyama and Nishinari, 1991) and the ninhydrin method (Ábrahám et al., 2010) were used to measure soluble sugars (SS) and proline (Pro), respectively.

### 2.5 Transcriptomic analysis

Total RNA was isolated from approximately 100 mg of flash-frozen root tissue using TRIzol^®^ reagent (Invitrogen, Carlsbad, CA, USA) following the manufacturer’s protocol. To eliminate potential genomic DNA contamination, the extracted RNA was treated with DNase I (Promega, USA). The quality and integrity of the RNA were then rigorously assessed (He et al., 2021). RNA quantity was determined with a Qubit RNA assay kit (Life Technologies, USA), while its integrity was evaluated using an RNA Nano assay kit on an Agilent Bioanalyzer (Agilent Technologies, USA). Only high-quality samples, defined by an RNA Integrity Number (RIN) ≥ 7.0 and a 28S/18S ratio ≥ 1.8, were selected for downstream sequencing. Library preparation commenced with the enrichment of mRNA from total RNA using poly-T oligo-attached magnetic beads. The purified mRNA was subsequently fragmented with a fragmentation buffer. First-strand cDNA synthesis was performed using random hexamer primers and M-MuLV Reverse Transcriptase, followed by second-strand synthesis with DNA Polymerase I and RNase H. These cDNA fragments then underwent end-repair, A-tailing, and ligation with sequencing adapters. Finally, the adapter-ligated fragments were enriched via PCR amplification to generate the final cDNA libraries. The quality and concentration of these libraries were verified using a Qubit 2.0 Fluorometer and an Agilent 2100 Bioanalyzer. The qualified libraries were then sequenced on an Illumina HiSeq 4000 platform to generate 150 bp paired-end reads (Trapnell et al., 2012).

Raw sequencing reads were subjected to a stringent quality control process. Adapter sequences, low-quality reads (Phred score < 20), and reads containing over 10% unknown nucleotides (N) were removed using SeqPrep and Sickle. The resulting high-quality clean reads were then aligned to the *N. tabacum* reference genome using TopHat (v2.0.0) (Trapnell et al., 2009). Gene expression levels were quantified and normalized as Fragments Per Kilobase of transcript per Million mapped reads (FPKM) using RSEM software. Differential expression analysis was performed between treatment groups (e.g., drought vs. control) and across different time points and varieties using the DESeq2 R package. Genes were identified as differentially expressed (DEGs) based on the criteria of an adjusted P-value < 0.05 and an absolute log2(fold change) ≥ 1. To elucidate the biological functions of these DEGs, functional annotation was conducted by aligning their sequences against public databases, including NCBI non-redundant protein (NR), Gene Ontology (GO), and Kyoto Encyclopedia of Genes and Genomes (KEGG), using BLAST+ (v2.9.0). Subsequently, GO and KEGG pathway enrichment analyses were carried out with Goatools and KOBAS, respectively. These analyses served to identify biological processes, molecular functions, and metabolic pathways that were significantly over-represented among the DEGs.

To identify significant temporal expression patterns, the Short Time-series Expression Miner (STEM) software (v1.3.13) was employed. Normalized FPKM values of DEGs from the time-course samples (0, 14, 21, 28, and 35 days) were clustered into a maximum of 50 model profiles. Profiles were considered statistically significant if their permutation test p-value was < 0.05, and these significant profiles were subjected to further GO and KEGG enrichment analysis. Furthermore, gene co-expression networks were constructed using the Weighted Gene Co-expression Network Analysis (WGCNA) R package. This analysis, performed after filtering out genes with low expression or variance, grouped genes with similar expression patterns into modules. The module eigengenes (the principal component of each module) were then correlated with physiological traits to identify key gene modules and hub genes significantly associated with the drought response.

### 2.6 Validation of RNA-Seq data with qRT-PCR

To validate the accuracy of the transcriptome data, real-time quantitative RT-PCR (qRT-PCR) experiments were performed. Total RNA was reverse transcribed into cDNA using a PrimeScript™ RT Reagent Kit (TaKaRa, Japan). qRT-PCR was performed on a LightCycler 480 II Real-Time PCR System (Roche, Switzerland) using SYBR Green I Master Mix (TaKaRa, Japan). Relative gene expression levels were calculated using the 2^^-^ΔΔCt^ method, with NtActin as the internal reference gene (Schmidt and Delaney, 2010). We randomly selected 15 DEGs for qPCR analysis, for which gene-specific primers were designed using Primer Premier v5.0 (**Supplementary Table S2**).

### 2.7 Statistical analysis

All measurements were performed in triplicate, and the data are presented as mean ± standard deviation (SD). Statistical significance of differences in root physiological parameters was assessed by one-way analysis of variance (ANOVA). *Post-hoc* comparisons were then conducted using Duncan’s multiple range test, with a significance level set at p < 0.05. All statistical analyses were performed using SPSS software (v24.0, SPSS Inc., Chicago, IL, USA).

## 3 Results

### 3.1 Effects of drought stress on root cell ultrastructure of *N. tabacum*

**Figure 1:**
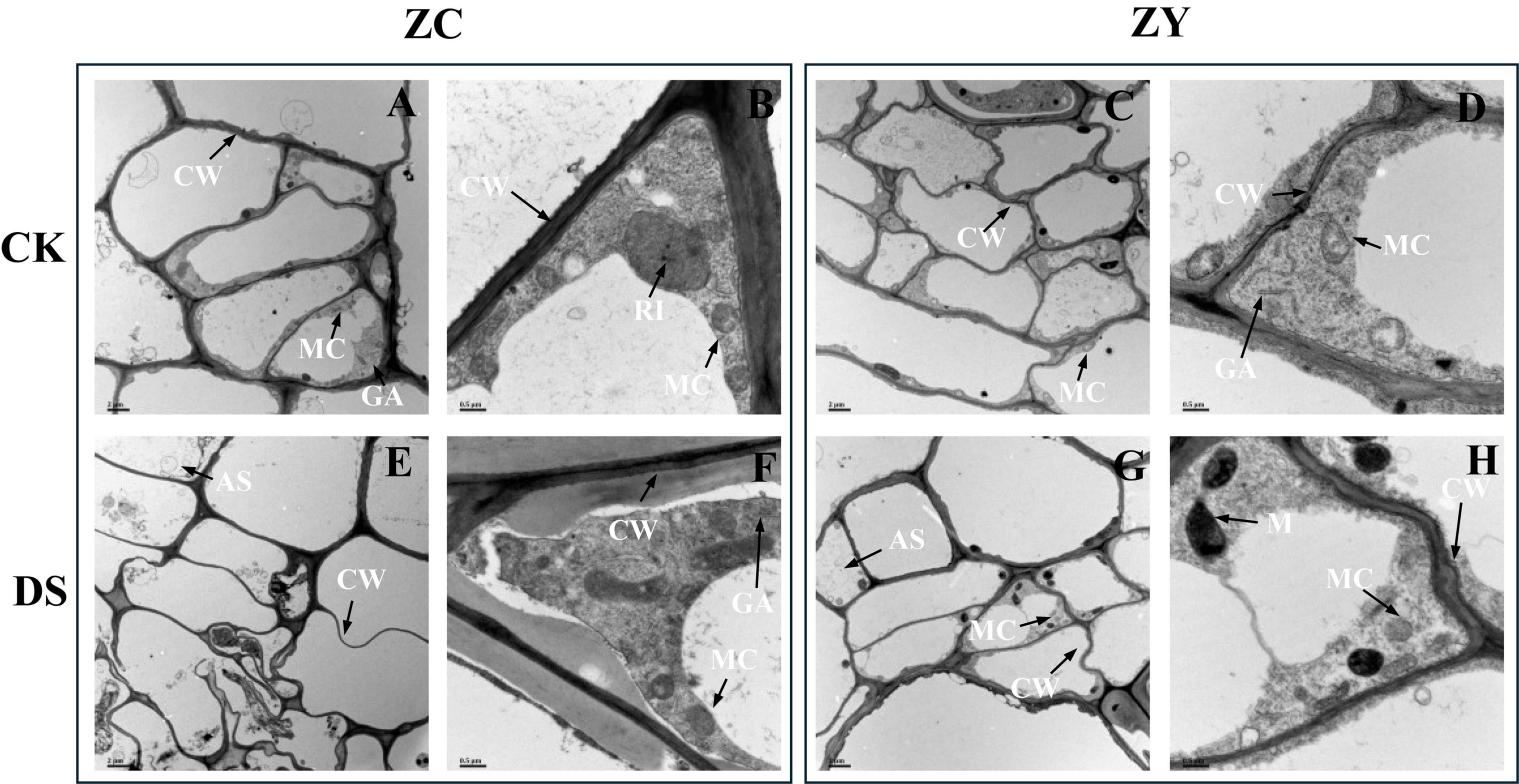
Ultrastructural changes of *Nicotiana tabacum* root cells under soil drought stress conditions observed via transmission electron microscopy. **(A, B)** ZC208 genotype under control (CK) conditions at 3 μm and 0.5 μm scales, respectively. **(C, D)** ZY100 genotype under control (CK) conditions at 3 μm and 0.5 μm scales, respectively. **(E, F)** ZC208 genotype under drought stress (DS) conditions at 3 μm and 0.5 μm scales, respectively. **(G, H)** ZY100 genotype under drought stress (DS) conditions at 3 μm and 0.5 μm scales, respectively. Abbreviations: ZC, ZC208 genotype; ZY, ZY100 genotype; CK, control; DS, drought stress; CW, cell wall; MIT, mitochondria; RI, ribosome; GA, Golgi apparatus; M, mucilage; AS, autolysosome-like structures.

We scanned the cell ultrastructures of the roots in different water treatments using TEM (**Figure 1**). Under prolonged drought stress (35 days), root cells exhibited distinct ultrastructural degradation compared to the intact organelles in control samples (**Figures 1A–D**). Specifically, mitochondria showed significant swelling, loss of matrix density, and the partial or complete disappearance of internal cristae (**Figures 1G–H**), while the Golgi apparatus cisternae became dilated and fragmented, losing their organized stacked arrangement (**Figure 1F**). The ‘decomposition of organelles’ was further evidenced by the presence of numerous autolysosome-like structures engulfing cytoplasmic debris and degraded organellar remnants (**Figures 1E, G**), alongside the accumulation of amorphous mucilage and apparent plasma membrane detachment (**Figure 1H**). These concrete morphological changes, particularly the breakdown of energy-producing and secretory organelles, provide a clear structural basis for the observed physiological decline under severe water deficit.

### 3.2 The level of oxidative damage on *N. tabacum* roots

**Figure 2:**
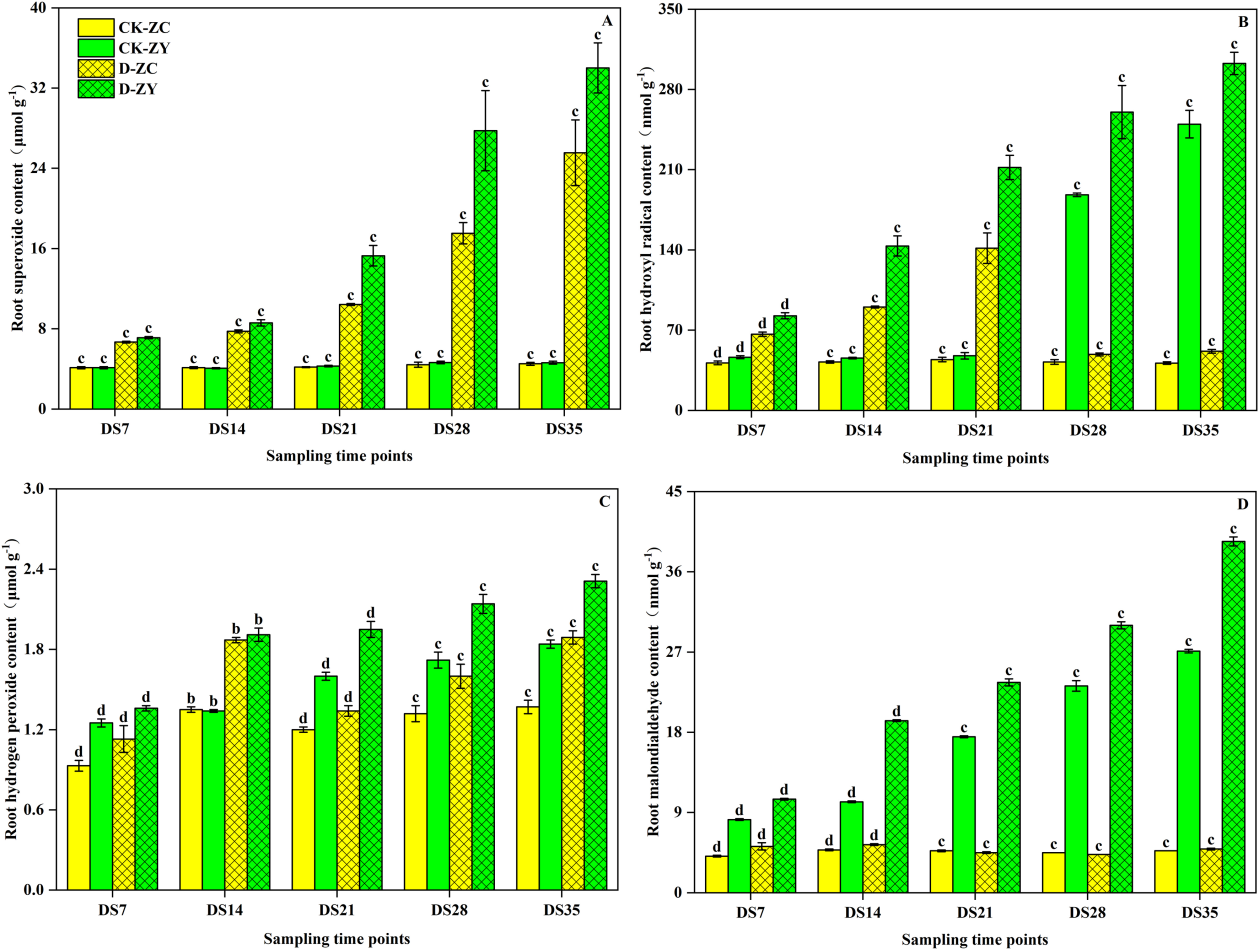
Effects of soil drought stress on the contents of superoxide **(A)**, hydroxyl radical **(B)**, hydrogen peroxide **(C)**, and malondialdehyde **(D)** in roots of *Nicotiana tabacum*. Vertical bars represent ± SD of the mean (n = 4); different letters on the SD bars indicate significant differences among the water treatments (*p* < 0.05).

The contents of O_2_^•^-^^, H_2_O_2_, ^•^OH, and MDA in *N. tabacum* roots under different water treatments are shown in **Figure 2**. Soil drought stress significantly enhanced the level of oxidative damage, as evidenced by increased contents of O_2_^•^-^^, H_2_O_2_, ^•^OH, and MDA. Furthermore, with increasing drought duration, the level of oxidative damage caused by drought stress was remarkably enhanced. Throughout the five stages of drought stress, the levels of O_2_^•^-^^, H_2_O_2_, ^•^OH and MDA in the DS-ZC treatment increased by 62.0–466.1%, 21.5–37.4%, 60.3–504.7%, and 97.5–478.5%, respectively, compared to the CK-ZC treatment (**Figures 2A–D**). Likewise, in the DS-ZY treatment, the levels of O_2_^•^-^^, H_2_O_2_, ^•^OH, and MDA rose by 72.8–636.6%, 9.2–42.6%, 78.0–486.2%, and 99.8–698.5%, respectively, when compared to the CK-ZY treatment (**Figures 2A–D**).

### 3.3 Effects of drought stress on the root biomass and activity of *N. tabacum*

**Figure 3:**
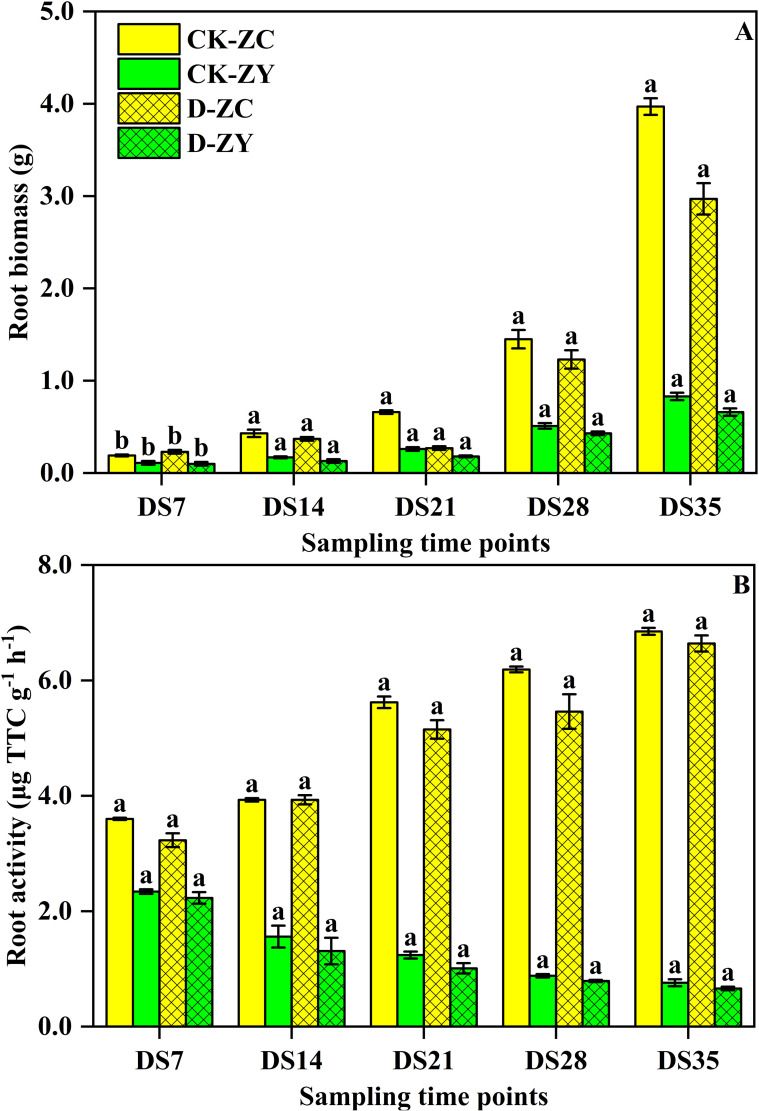
Effects of soil drought stress on root activity **(A)** and biomass **(B)** of *Nicotiana tabacum*. Vertical bars represent ± SD of the mean (n = 3); different letters on the SD bars indicate significant differences among the soil water treatments (p < 0.05).

The dynamic changes in root biomass (RB) and root activity (RA) in *N. tabacum* seedlings alongside the duration of drought stress are shown in **Figure 3**. Both RB av A were significantly reduced by drought stress, and the values of these parameters in the DS-ZY treatment were lower than those in the DS-ZC treatment. Across the five drought stress stages, the RB in the DS-ZC treatment decreased by 42.1–79.1% compared to the CK-ZC treatment, while the RB in the DS-ZY treatment decreased by 33.3–77.8% compared to the CK-ZY treatment. With increasing drought stress stage, the RA in the DS-ZC treatment decreased from 2.34 μg TTC g^−1^ h^−1^ to 0.76 μg TTC g^−1^ h^−1^, while the RA in the DS-ZY treatment decreased from 2.23 μg TTC g^−1^ h^−1^ to 0.66 μg TTC g^−1^ h^−1^1.

### 3.4 Dynamic responses of the root system architecture of *N. tabacum* to drought stress

Soil drought stress induced significant alterations in RSA. Analysis of the data presented in **Table 1** revealed that drought stress had a pronounced effect on the majority of measured RSA parameters (*p* < 0.05). Specifically, with the progression of drought stress, key root growth indicators including RL, PA, SA in both cultivars (DS-ZC and DS-ZY) exhibited a continuous declining trend, reaching their minimum values at the 35th day of stress. Compared to their respective controls (CK), these parameters were significantly reduced under drought conditions. Conversely, two key indicators reflecting root morphology, SRSA and SRV, displayed an opposing trend in response to drought. The values of SRSA and SRV increased significantly in both the DS-ZC and DS-ZY treatments, suggesting that roots may adapt to water-limited conditions by altering their construction strategy. Regarding inter-varietal differences, although drought stress significantly impacted the RSA of both cultivars, the magnitude of these changes varied. For instance, under drought conditions, RL of the tolerant variety ZC208 decreased by 50.2% compared to its control, a more pronounced reduction than the 41.7% decrease observed in the sensitive variety ZY100. However, in terms of compensatory increases, ZY100 showed a greater enhancement in both SRSA (169.5% increase) and SRV (181.5% increase) compared to ZC208 (148.2% and 159.3% increases, respectively).

**Table 1:** Phenotypic variation of *Nicotiana tabacum* root-related traits under drought and well-watered conditions.

Drought days	Treatments	RL (cm)	PA (cm^2^)	SA (cm^2^)	AD (mm)	LPA (cm m^-3^)	RV (cm^3^)	SRL (cm g^-1^)	SRSA (cm^2^ g^-1^)	SRV (cm^3^ g^-1^)	TIP	FOR	CRO
DS7	CK-ZC	714.4 ± 28.2 a	62.0 ± 6.9 a	196.9 ± 22.0 a	1.09 ± 0.03 a	681.4 ± 26.7 a	3.94 ± 0.17 a	3772.3 ± 346.4 a	1042.5 ± 170.2 a	20.8 ± 2.0 a	3305 ± 276 b	1078 ± 66 a	94 ± 2 a
	D-ZC	318.7 ± 1.3 c	21.2 ± 0.9 c	66.2 ± 0.9 c	0.83 ± 0.03 b	320.2 ± 2.1 c	1.15 ± 0.07 c	2844.5 ± 360.3 b	591.7 ± 80.5 b	10.3 ± 1.3 bc	1440 ± 118 c	580 ± 10 c	36 ± 4 b
	CK-ZY	563.2 ± 19.9 b	38.2 ± 3.3 b	109.8 ± 1.8 b	0.51 ± 0.04 c	562.3 ± 19.8 b	1.74 ± 0.08 b	2454.4 ± 124.1 b	478.6 ± 29.3 b	7.6 ± 0.9 c	4160 ± 252 a	780 ± 15 b	30 ± 3 c
	D-ZY	275.7 ± 4.0 d	21.7 ± 0.6 c	66.9 ± 1.6 c	0.42 ± 0.03 d	273.7 ± 3.2 d	1.32 ± 0.03 c	2707.5 ± 208.8 b	657.3 ± 101.3 b	12.9 ± 1.6 b	662 ± 5 d	361 ± 2 d	18 ± 1 d
DS14	CK-ZC	1087.9 ± 28.1 a	69.7 ± 4.9 a	225.7 ± 7.1 a	0.64 ± 0.06 a	1085.7 ± 27.7 a	3.28 ± 0.15 b	2561.8 ± 192.3 b	533.5 ± 70.6 b	7.7 ± 0.4 b	5115 ± 177 b	2143 ± 339 a	151 ± 3 b
	D-ZC	512.9 ± 2.5 d	30.3 ± 0.8 b	94.5 ± 1.1 b	0.35 ± 0.03 b	512.2 ± 2.1 d	1.44 ± 0.08 c	3086.9 ± 208.2 b	568.7 ± 38.4 b	8.7 ± 0.7 b	2005 ± 34 c	925 ± 10 c	88 ± 1 d
	CK-ZY	1010.5 ± 40.3 b	73.7 ± 5.1 a	221.0 ± 29.0 a	0.72 ± 0.12 a	1007.8 ± 38.9 b	4.01 ± 0.19 a	2713.9 ± 216.1 b	592.7 ± 76.0 b	10.8 ± 0.9 a	6533 ± 348 a	2285 ± 201 a	198 ± 11 a
	D-ZY	639.2 ± 4.9 c	35.2 ± 0.4 b	110.7 ± 1.5 b	0.44 ± 0.05 b	639.6 ± 5.7 c	1.55 ± 0.04 c	5099.2 ± 661.8 a	882.6 ± 111.0 a	12.4 ± 1.8 a	2035 ± 9 c	1394 ± 83 b	134 ± 2 c
DS21	CK-ZC	1395.2 ± 7.7 a	101.3 ± 4.1 a	318.1 ± 15.6 a	0.73 ± 0.03 a	1395.3 ± 7.8 a	6.28 ± 0.48 a	2104.5 ± 55.6 d	480.4 ± 38.2 c	9.5 ± 0.8 b	5937 ± 22 a	3522 ± 110 a	434 ± 11 a
	D-ZC	697.8 ± 8.9 c	35.0 ± 0.2 d	109.8 ± 0.5 d	0.31 ± 0.01 c	697.5 ± 8.8 c	1.37 ± 0.01 c	2690.6 ± 150.5 c	423.6 ± 26.5 c	5.3 ± 0.4 c	3978 ± 127 c	1391 ± 83 c	120 ± 8 b
	CK-ZY	1391.9 ± 10.4 a	88.4 ± 0.9 b	278.6 ± 1.4 b	0.66 ± 0.02 b	1390.8 ± 10.0 a	4.45 ± 0.02 b	5232.0 ± 329.0 a	1047.2 ± 62.5 a	16.7 ± 1.0 a	4850 ± 7 b	2761 ± 2 b	422 ± 7 a
	D-ZY	798.2 ± 2.2 b	42.1 ± 0.2 c	134.2 ± 2.4 c	0.33 ± 0.02 c	798.2 ± 2.2 b	1.75 ± 0.02 c	4521.7 ± 160.8 b	760.4 ± 29.8 b	9.9 ± 0.3 b	3797 ± 10 d	1491 ± 12 c	119 ± 2 b
DS28	CK-ZC	1886.0 ± 23.7 a	107.4 ± 6.3 a	338.2 ± 19.8 a	0.57 ± 0.05 a	1886.4 ± 23.1 a	4.81 ± 0.29	1301.4 ± 81.2 c	233.7 ± 25.9 c	3.3 ± 0.4 c	9945 ± 171 a	4504 ± 384 a	475 ± 11 a
	D-ZC	985.0 ± 3.0 d	57.3 ± 1.0 d	178.4 ± 1.8 c	0.43 ± 0.02 b	978.2 ± 4.9 d	2.56 ± 0.04 c	1935.8 ± 112.2 b	350.5 ± 19.2 b	5.0 ± 0.3 b	5439 ± 11 c	2455 ± 15 b	321 ± 2 b
	CK-ZY	1666.6 ± 32.5 b	96.7 ± 0.5 b	306.0 ± 5.4 b	0.58 ± 0.01 a	1666.6 ± 32.6 b	4.39 ± 0.06 b	1366.4 ± 141.9 c	250.6 ± 20.4 c	3.6 ± 0.3 c	9205 ± 346 b	4538 ± 400 a	462 ± 15 a
	D-ZY	1125.9 ± 4.0 c	68.0 ± 0.6 c	193.4 ± 2.1 c	0.40 ± 0.02 b	1125.0 ± 2.8 c	2.65 ± 0.06 c	2644.4 ± 156.7 a	454.4 ± 30.0 a	6.2 ± 0.5 a	4395 ± 3 d	2665 ± 20 b	353 ± 8 b
DS35	CK-ZC	2449.2 ± 13.5 a	134.4 ± 3.6 a	414.8 ± 2.0 a	0.56 ± 0.02 a	2437.3 ± 2.5 a	5.60 ± 0.04 a	616.7 ± 17.0 c	104.4 ± 2.5 c	1.4 ± 0.0 c	8333 ± 8 b	6908 ± 82 a	948 ± 4 a
	D-ZC	1219.8 ± 45.7 c	68.3 ± 8.1 c	216.4 ± 25.7 c	0.34 ± 0.03 b	1219.8 ± 14.4 c	3.05 ± 0.31 c	1464.2 ± 25.8 ± b	259.2 ± 19.9 b	3.7 ± 0.2 b	6836 ± 273 c	3043 ± 151 c	297 ± 2 d
	CK-ZY	2023.3 ± 2.4 b	106.9 ± 0.56 b	337.6 ± 2.8 b	0.56 ± 0.03 a	2022.2 ± 2.0 b	4.46 ± 0.05 b	682.8 ± 40.1 c	114.0 ± 7.2 c	1.5 ± 0.1 c	8772 ± 61 a	5870 ± 57 b	737 ± 8 b
	D-ZY	1179.3 ± 4.5 c	64.6 ± 2.7 c	202.2 ± 8.4 c	0.34 ± 0.02 b	1179.3 ± 4.5 d	2.79 ± 0.24 c	1790.2 ± 92.8 a	307.1 ± 23.8 a	4.2 ± 0.5 a	5798 ± 206 d	2686 ± 26 d	337 ± 7 c

AD, average diameter; CRO; crossings; FOR; forks; LPA, length per volume; PA, project area; RV, root volume; RL, root length; SA, surface area; SRL, specific root length; SRSA, specific root surface area; SRV, specific root volume; TIP, tips. Values are the mean of four replicates. Different letters in the same column indicate signiﬁcant differences (p < 0.05) between different treatment groups according Duncan’s test.

### 3.5 Effects of drought stress on antioxidant enzyme activities and soluble sugar and proline contents in *N. tabacum* roots

The activities of antioxidant enzymes in the roots of *N. tabacum* seedlings exposed to drought stress are shown in **Table 2**. Drought stress led to a significant increase in the activities of SOD, POD, and CAT. Furthermore, with increasing drought duration, the values of these enzymes showed a remarkable increase, with the highest values found at the 35th drought stress stage. At this stage, the activities of SOD, POD, and CAT in the DS-ZC treatment increased by 76.5, 453.1, and 29.0%, respectively, compared to the CK-ZC treatment. Compared to the CK-ZY treatment, the activities of SOD, POD, and CAT in the DS-ZC treatment increased by 52.3, 386.4, and 18.7%, respectively.

**Table 2:** Effects of drought stress on the activities of antioxidant enzymes and contents of proline and soluble sugar in *Nicotiana tabacum* roots.

Drought days	Treatments	SOD activity (U g^-1^)	POD activity (U g^-1^)	CAT activity (U g^-1^)	Pro content (μg g^-1^)	SS content (mg g^-1^)
DS7	CK-ZC	206.0 ± 7.4 c	400.5 ± 3.6 c	233.7 ± 3.3 b	31.2 ± 1.1 c	1.09 ± 0.18 c
	D-ZC	262.3 ± 12.5 a	1509.4 ± 155.3 a	262.1 ± 8.5 a	38.4 ± 1.7 b	0.86 ± 0.05 d
	CK-ZY	183.7 ± 4.3 d	388.6 ± 5.9 c	192.5 ± 7.0 c	29.6 ± 1.5 c	1.76 ± 0.04 a
	D-ZY	241.4 ± 8.4 b	1127.0 ± 8.6 b	247.2 ± 17.4 ab	43.0 ± 1.6 a	1.26 ± 0.05 b
DS14	CK-ZC	222.3 ± 9.0 c	416.8 ± 3.0 c	254.1 ± 3.5 b	33.9 ± 1.4 c	2.14 ± 0.04 c
	D-ZC	309.0 ± 7.6 a	1807.4 ± 25.6 a	304.5 ± 7.5 a	43.7 ± 1.7 b	1.29 ± 0.04 d
	CK-ZY	193.4 ± 5.8 d	379.4 ± 14.2 c	137.1 ± 9.0 d	31.8 ± 1.5 c	3.60 ± 0.08 a
	D-ZY	274.5 ± 6.8 b	1491.4 ± 63.6 b	172.4 ± 7.4 c	48.4 ± 1.7 a	2.82 ± 0.04 b
DS21	CK-ZC	211.6 ± 2.9 c	429.2 ± 3.0 c	258.7 ± 7.4 b	34.4 ± 1.2 d	2.64 ± 0.12 c
	D-ZC	364.1 ± 9.5 a	2554.9 ± 33.2 a	351.3 ± 2.5 a	47.7 ± 1.9 b	1.25 ± 0.06 d
	CK-ZY	188.4 ± 9.3 d	399.0 ± 16.0 c	152.7 ± 5.8 d	37.2 ± 1.4 c	6.08 ± 0.05 a
	D-ZY	300.3 ± 1.8 b	2110.3 ± 131.6 b	196.5 ± 5.1 c	55.6 ± 0.7 a	3.18 ± 0.06 b
DS28	CK-ZC	224.4 ± 12.1 c	436.2 ± 10.6 c	212.0 ± 11.5 b	34.6 ± 1.1 d	3.24 ± .05 c
	D-ZC	392.0 ± 3.2 a	2698.7 ± 44.5 a	278.5 ± 4.8 a	51.3 ± 1.0 b	1.77 ± 0.07 d
	CK-ZY	211.6 ± 11.2 c	476.0 ± 5.4 c	136.4 ± 17.3 d	37.2 ± 1.0 c	9.73 ± 0.10 a
	D-ZY	351.5 ± 16.1 b	2201.0 ± 12.0 b	182.1 ± 3.5 c	57.8 ± 1.8 a	4.71 ± 0.09 b
DS35	CK-ZC	231.4 ± 6.9 c	526.2 ± 14.8 c	172.8 ± 8.3 b	33.3 ± 1.0 c	5.65 ± 0.13 b
	D-ZC	408.3 ± 7.1 a	2910.2 ± 40.2 a	222.9 ± 2.6 a	57.2 ± 2.5 b	2.35 ± 0.03 d
	CK-ZY	241.1 ± 16.0 c	467.5 ± 11.5 d	133.3 ± 3.3 d	34.4 ± 1.1 c	11.78 ± 0.49 a
	D-ZY	367.1 ± 8.5 b	2274.2 ± 27.3 b	158.3 ± 2.4 c	65.3 ± 1.2 a	4.23 ± 0.11 c

APX, ascorbate peroxidase; CAT, Catalase; SOD, superoxide dismutase; SS, soluble sugar; POD, peroxidase; Pro, proline. Values are the mean of four replicates. Different letters in the same column indicate signiﬁcant differences (*p* < 0.05) between different treatment groups according Duncan’s test.

The Pro contents in the roots were also significantly enhanced when the plants suffered soil water deficit, while the SS contents were remarkably reduced (**Table 2**). Across the five drought stress stages, compared to the CK-ZC treatment, the contents of SS decreased by 21.3–58.4% and Pro increased by 23.2–71.5% in the DS-ZC treatment. Similarly, compared to the CK-ZY treatment, the contents of SS decreased by 28.5–64.1% and Pro increased by 45.4–89.5% in the DS-ZY treatment.

### 3.6 Transcriptomic changes of *N. tabacum* roots under drought conditions

To investigate the transcriptional changes in *N. tabacum* roots in response to drought, we compared the expression profiles of well-watered and drought-stressed roots from both varieties at five different time points: 7, 14, 21, 28, and 35 days. After data filtering and quality control, we obtained a total of 2.93 billion clean reads from 60 root samples (**Supplementary Table S3**). The Q20 and Q30 statistics for the clean reads exceeded 90.0 and 96.7%, respectively, with an average GC content of 42.2%. The sequencing quality was high, and the alignment rate with the reference genome was satisfactory, indicating reliable sequencing results. Ultimately, 66,812 genes were identified by mapping to the reference genome for further analysis.

To distinguish the differences in gene transcription levels between different water treatments and plant species at the five different time points, we conducted principal component analysis (PCA) (**Supplementary Figure 2**). The results revealed that the 12 samples at the 7th, 14th, 21st, 28th, and 35th time points were clearly separated into four groups, suggesting that the overall transcriptome profile under the same treatment had similar gene expression and there were remarkable differences between CK-ZC, DS-ZC, CK-ZY, and DS-ZY treatments. In addition, we conducted PCA to determine the differences between the five drought stress time points in the same treatment. As illustrated in **Supplementary Figure 3**, there were remarkable differences, suggesting that root transcription showed different expression levels at different drought stress time points.

**Figure 4:**
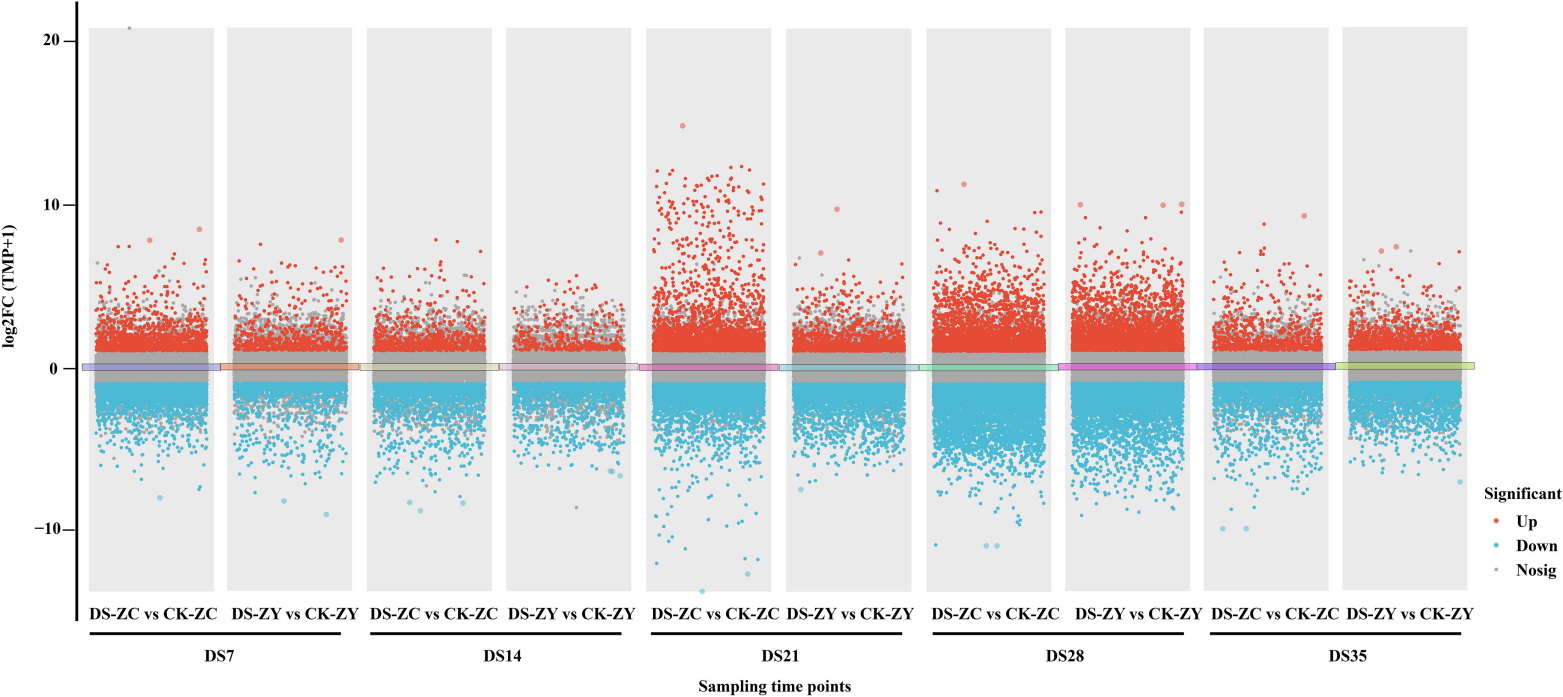
Manhattan of differentially expressed genes in roots of *Nicotiana tabacum* under drought stress compared with control samples. ZC, ZC208 genotype; ZY, ZY100 genotype; CK, well-watered; DS, drought stress. DS7, DS14, DS21, DS28 and DS35 indicate the plants suffered drought stress for 7, 14, 21, 28 and 35 days, respectively.

We then conducted a differential expression analysis to identify the genes that exhibited significant changes in the roots under varying levels of drought stress compared to the control (**Figure 4**; **Supplementary Figure 4**). Based on the DESeq2 analysis results, we identified 1601 DEGs in the DS-ZC vs. CK-ZC group, with 587 upregulated and 1014 downregulated genes at the DS7 stage. This increased to 2183 DEGs (736 upregulated and 1447 downregulated) at the DS14 stage, 6802 (2625 upregulated and 4177 downregulated) at the DS21 stage, 7511 (3205 upregulated and 4306 downregulated) at the DS28 stage, and 3483 (1200 upregulated and 2283 downregulated) at the DS35 stage. In comparison, the DS-ZY treatment resulted in 2212 DEGs (699 upregulated and 1513 downregulated) at the DS7 stage, 1414 (339 upregulated and 1075 downregulated) at the DS14 stage, 4190 (1118 upregulated and 3072 downregulated) at the DS21 stage, 8132 (2853 upregulated and 5279 downregulated) at the DS28 stage, and 2934 (989 upregulated and 1945 downregulated) at the DS35 stage. This pattern suggests an increasing transcriptomic response with an intensifying water deficit. Notably, the number of upregulated DEGs was consistently lower than that of downregulated DEGs across all drought stress stages.

### 3.7 Functional enrichment analysis of identified DEGs

**Figure 5:**
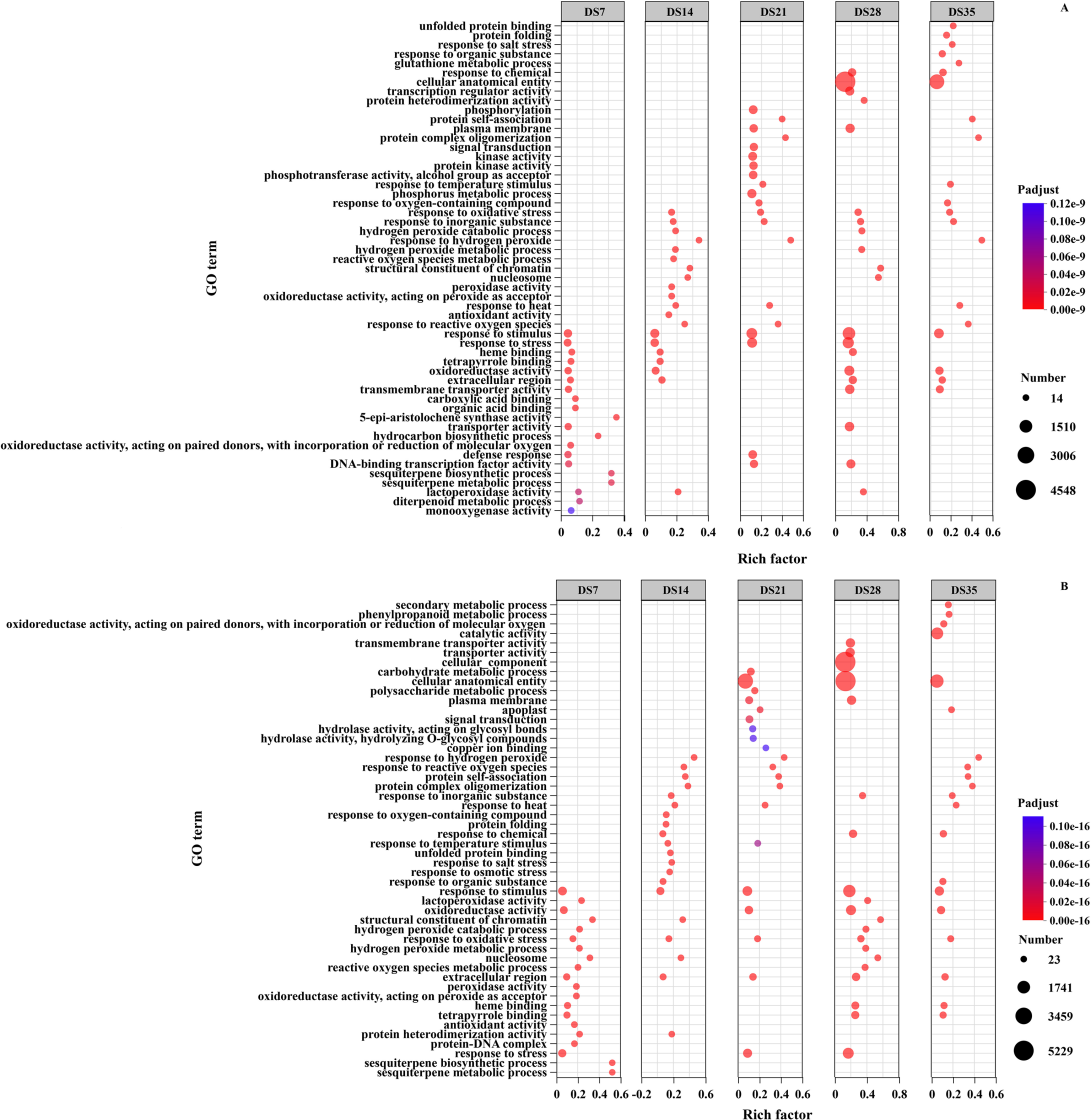
Go enrichment analysis of differentially expressed genes in DS-ZC vs CK-ZC **(A)** and DS-ZY vs CK-ZY **(B)** groups. ZC, ZC208 genotype; ZY, ZY100 genotype; CK, well-watered; DS, drought stress. DS7, DS14, DS21, DS28 and DS35 indicate the plants suffered drought stress for 7, 14, 21, 28 and 35 days, respectively.

To understand the biological processes of *N. tabacum* in response to drought stress, we identified GO terms related to biological processes, cellular components, and molecular functions (**Figure 5**). In the DS-ZC vs. CK-ZC group, the GO enrichment analysis indicated that the most significant terms included “5-epi-aristolochene synthase activity” (GO:0102698), “sesquiterpene biosynthetic process” (GO:0051762), and “sesquiterpene metabolic process” (GO:0051761) during the 7th drought period. In the 14th drought period, significant terms included “response to hydrogen peroxide” (GO:0042542) and “structural constituent of chromatin” (GO:0030527). The 21st drought period highlighted “response to hydrogen peroxide” (GO:0042542), “protein self-association” (GO:0043621), and “protein complex oligomerization” (GO:0051259). In the 28th drought period, the significant terms were “structural constituent of chromatin” (GO:0030527) and “nucleosome” (GO:0000786), while the 35th period included “response to hydrogen peroxide” (GO:0042542), “protein complex oligomerization” (GO:0051259), and “protein self-association” (GO:0043621). For the DS-ZY vs. CK-ZY group, the most significant GO terms included “sesquiterpene biosynthetic process” (GO:0051762) and “sesquiterpene metabolic process” (GO:0051761) during the 7th drought period, followed by “response to hydrogen peroxide” (GO:0042542) in the 14th drought period. The 21st drought period also showed significant terms such as “response to hydrogen peroxide” (GO:0042542), “protein self-association” (GO:0043621), and “protein complex oligomerization” (GO:0051259). In the 28th drought period, the significant terms were “structural constituent of chromatin” (GO:0030527) and “nucleosome” (GO:0000786), while the 35th period included “response to hydrogen peroxide” (GO:0042542), “protein complex oligomerization” (GO:0051259), and “response to reactive oxygen species” (GO:0000302).

**Figure 6:**
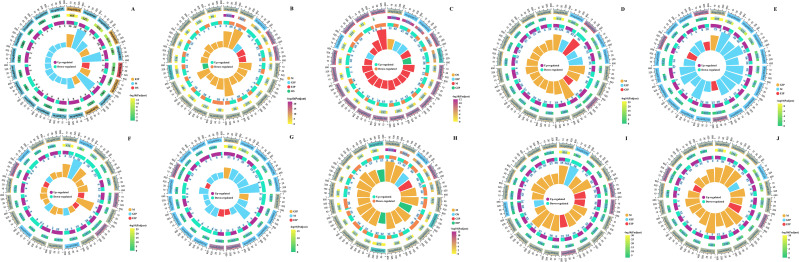
Multidimensional enrichment plot of top 20 differentially expressed genes in different comparison groups of *Nicotiana tabacum* leaves in different comparison groups. **(A–E)**: The KEGG enrichment of differentially expressed genes of DS-ZC vs CK-ZC group in DS7, DS14, DS21, DS28 and DS35 stage. **(F–J)**: The KEGG enrichment of differentially expressed genes of DS-ZY vs CK-ZY group in DS7, DS14, DS21, DS28 and DS35 stage. ZC, ZC208 genotype; ZY, ZY100 genotype; CK, well-watered; DS, drought stress. DS7, DS14, DS21, DS28 and DS35 indicate the plants suffered drought stress for 7, 14, 21, 28 and 35 days, respectively. EIP, Environmental Information Processing; GIP, Genetic Information Processing; M, Metabolism; OS, Organismal Systems.

To further investigate the potential function of DEGs in response to drought stress, we performed a KEGG enrichment analysis (**Figure 6**). For the DS-ZC vs. CK-ZC group, the most enriched pathways were “sesquiterpenoid and triterpenoid biosynthesis” (map00909), “diterpenoid biosynthesis” (map00904), and “taurine and hypotaurine metabolism” (map00430) in the 7th drought period, “phenylpropanoid biosynthesis” (map00940), “sesquiterpenoid and triterpenoid biosynthesis” (map00909), and “taurine and hypotaurine metabolism” (map00430) in the 14th drought period, “stilbenoid, diarylheptanoid and gingerol biosynthesis” (map00945) and “betalain biosynthesis” (map00965) in the 21st drought period, “DNA replication” (map03030), “phenylpropanoid biosynthesis” (map00940), and “taurine and hypotaurine metabolism” (map00430) in the 28th drought period, and “glutathione metabolism” (map00052), “biosynthesis of various plant secondary metabolites” (map00999), and “taurine and hypotaurine metabolism” (map00430) in the 35th drought period (**Figure 6A**). For the DS-ZY vs. CK-ZY group, the most significantly enriched pathways were “DNA replication” (map03030) and “taurine and hypotaurine metabolism” (map00430) in the 7th drought period, “taurine and hypotaurine metabolism” (map00430) and “monoterpenoid biosynthesis” (map00902) in the 14th drought period, “phenylpropanoid biosynthesis” (map00940), “starch and sucrose metabolism” (map00500), and “biosynthesis of various plant secondary metabolites” (map00999) in the 21st drought period, “DNA replication” (map03030), “taurine and hypotaurine metabolism” (map00430), and “phenylpropanoid biosynthesis” (map00940) in the 28th drought period, and “glycosaminoglycan degradation” (map00531), “biosynthesis of various plant secondary metabolites” (map00999), and “diterpenoid biosynthesis” (map00904) in the 35th drought period (**Figure 6B**).

### 3.8 Time-course transcriptome analysis of drought-stressed *N. tabacum* roots

**Figure 7:**
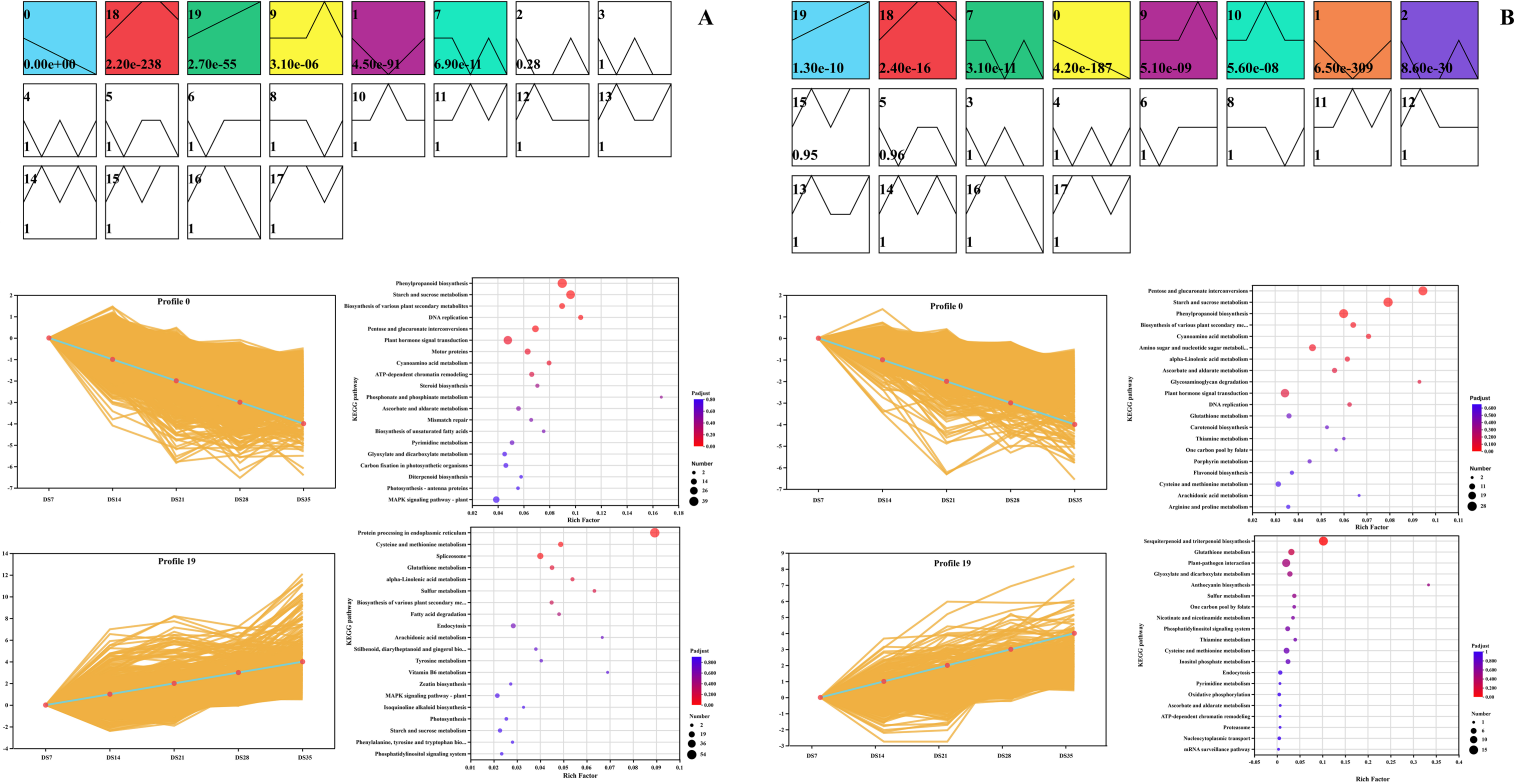
Cluster analysis revealed distinct gene expression patterns of DS-ZC vs CK-ZC **(A)** and DS-ZY vs CK-ZY **(B)** groups in *Nicotiana tabacum* seedlings in response to drought treatments. The line charts show the dynamic changes of differentially expressed genes in profile 0 and 19. The bubble plot present the KEGG enrichment of differentially expressed genes in profile 0 and 19. DS7, DS14, DS21, DS28 and DS35 indicate the plants suffered drought stress for 7, 14, 21, 28 and 35 days, respectively.

We utilized the STEM software to group genes with similar transcriptional changes across different time points, with the goal of systematically identifying the dynamic expression patterns of DEGs following drought stress exposure. As shown in **Figure 7**, a total of six expression profiles were identified for the ZC208 genotype and eight for the ZY100 genotype, organized by the number of DEGs (p < 0.05). For the ZC208 genotype, the profiles in ascending order were 0, 1, 7, 9, 18, and 19, corresponding to 1705, 1464, 628, 1493, and 905 DEGs, respectively (**Figure 7A**). In the ZY100 genotype, the profiles in ascending order were 0, 1, 2, 7, 9, 10, 18, and 19, with the number of DEGs being 1265, 1971, 704, 575, 558, 471, 772, and 662, respectively (**Figure 7B**). Notably, the DEGs in profile 0 for ZC208 were downregulated, while those in profile 19 were upregulated with increasing processing time. Similarly, in ZY100, profiles 0 and 19 also showed upregulation and downregulation, respectively, with the progression of drought time points.

We subjected the DEGs in profiles 0 and 19 for the ZC208 and ZY100 genotypes to KEGG enrichment analysis. In the ZC208 genotype, the DEGs in profile 0 were primarily enriched in the pathways of “phenylpropanoid biosynthesis” (map00940), “starch and sucrose metabolism” (map00500), and “plant hormone signal transduction” (map04075) (**Figure 7A**). Conversely, profile 19 showed significant enrichment in the pathway of “protein processing in the endoplasmic reticulum” (**Figure 7A**). For the ZY100 genotype, the DEGs in profile 0 were mainly enriched in “pentose and glucuronate interconversions” (map00040), “starch and sucrose metabolism” (map00500), “phenylpropanoid biosynthesis” (map00940), and “plant hormone signal transduction” (map04075) (**Figure 7B**). In profile 19, the DEGs were predominantly enriched in the pathway of “sesquiterpenoid and triterpenoid biosynthesis” (map04141) (**Figure 7B**).

### 3.9 Impact of drought stress on the plant hormone signaling pathway

**Figure 8:**
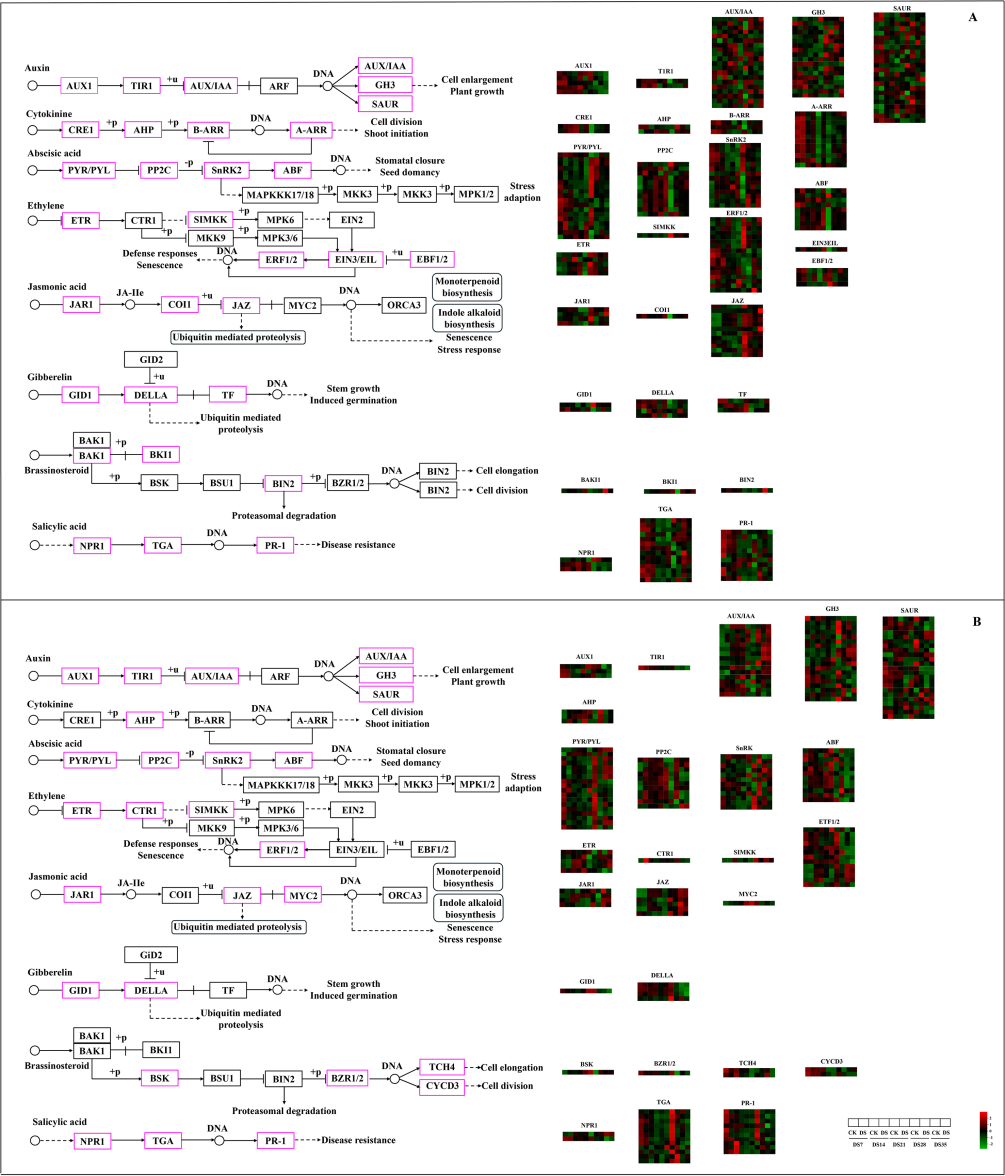
KEGG enriched differentially expressed genes in DS-ZC vs CK-ZC **(A)** and DS-ZY vs CK-ZY **(B)** groups involved in plant hormone signaling pathway. The expression levels of genes are shown from green to red (low to high) in the comparisons of CS72 vs. CK, respectively. The scales are generated using log2(fold change) values. Genes marked with pink color in the border of matrix represent significant changes under drought stress (*p* < 0.05).

Phytohormones are crucial for numerous developmental processes and responses to environmental stimuli. The transcriptome data was analyzed to further explore the relationship between drought stress and the plant hormone signaling pathway (**Figure 8**). A total of 233 (DS-ZC vs. CK-ZC) and 192 (DS-ZY vs. CK-ZY) genes were enriched in the plant hormone signal transduction pathway, with auxin, abscisic acid, ethylene, jasmonic acid, and salicylic acid signaling pathways being highly represented. The genes related to different plant hormone signaling pathways varied in expression patterns in ZC208 and ZY100, as well as in different drought time points, indicating differential responses to *N. tabacum* genotypes and drought strength. For example, in the DS-ZC vs. CK-ZC group, the genes related to AUX/IAA were upregulated in both DS7 and DS35 time points, and upregulated or downregulated in DS14, DS21, and DS28 time points (**Figure 8A**). In the DS-ZY vs. CK-ZY group, the DEGs related to AUX/IAA were upregulated in DS7, DS28, and DS35 time points, and upregulated or downregulated in DS14 and DS21 time points (**Figure 8B**). For the abscisic acid biosynthesis pathway of the ZC208 genotype, the genes related to PYR/PYL were significantly downregulated in the DS7, DS14, and DS35 time points, and upregulated or downregulated in DS21 and DS28 time points (**Figure 8A**). For the ZY100 genotype, the genes related to PYR/PYL were upregulated or downregulated in the DS21 time point but significantly downregulated in the other four time points (**Figure 8B**).

### 3.10 Starch and sucrose metabolism induced by drought stress

**Figure 9:**
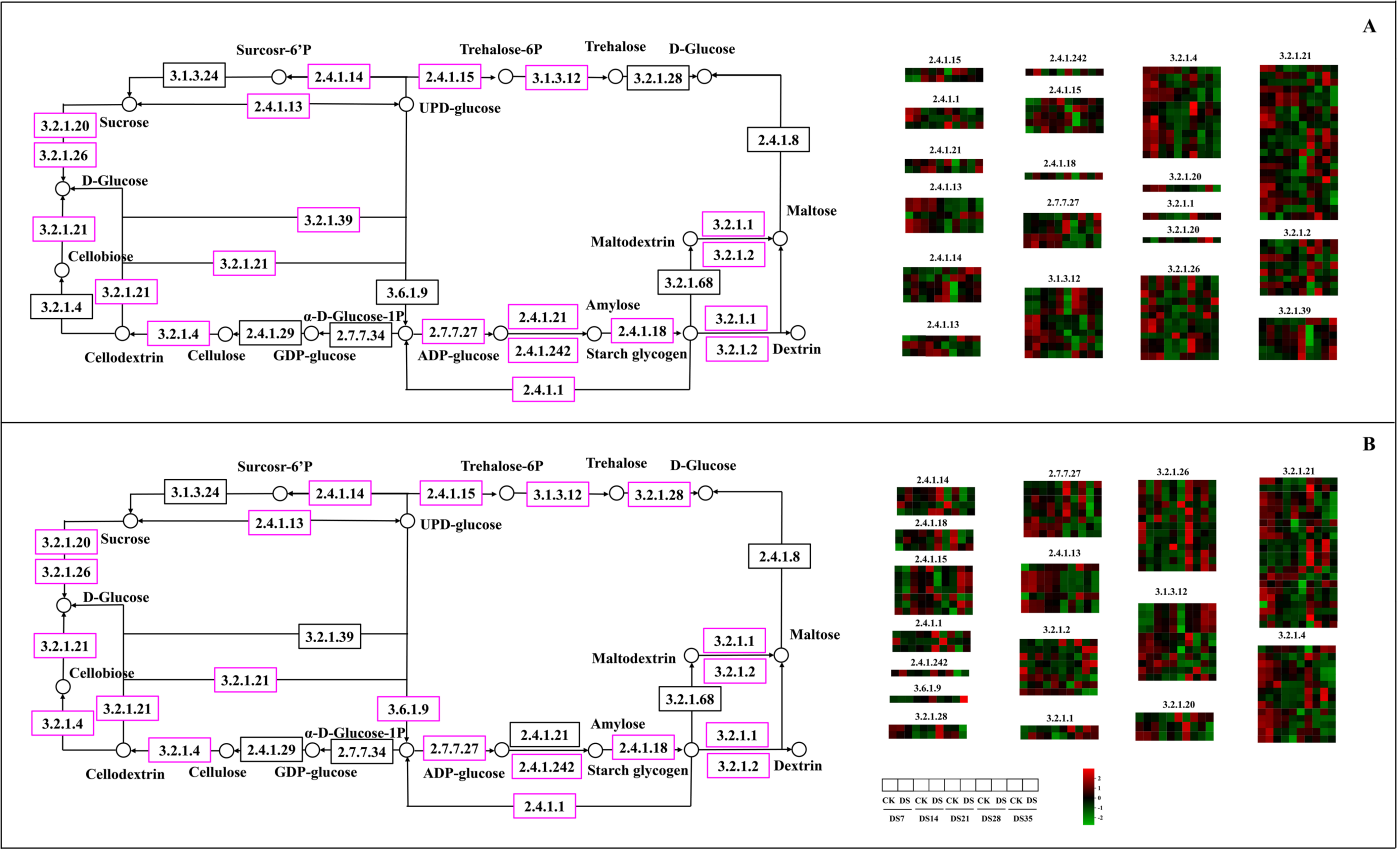
KEGG enriched differentially expressed genes in DS-ZC vs CK-ZC **(A)** and DS-ZY vs CK-ZY **(B)** groups involved in starch and sucrose metabolism. The expression levels of genes are shown from green to red (low to high) in the comparisons of CS72 vs. CK, respectively. The scales are generated using log2(fold change) values. Genes marked with pink color in the border of matrix represent significant changes under drought stress (*p* < 0.05).

The expression levels of genes involved in starch and sucrose metabolism are illustrated in **Figure 9**. Drought stress notably affected the expression of the majority of genes within these metabolic pathways. In the DS-ZC vs. CK-ZC group, the changed genes were mainly related to endoglucanase (EC3.2.1.4), trehalose 6-phosphate phosphatase (EC3.1.3.12), β-fructofuranosidase (IVV, EC3.2.1.26), β-glucosidase (EC3.2.1.21), and β-amylase (EC3.2.1.2). In the DS-ZY vs. CK-ZY group, the genes related to glucose-1-phosphate adenylyltransferase (EC2.7.27), trehalose 6-phosphate synthase (EC2.4.1.15), β-amylase (EC3.2.1.2), β-fructofuranosidase (EC3.2.1.26), trehalose 6-phosphate phosphatase (EC3.1.3.12), endoglucanase (EC3.2.1.4), and β-glucosidase (EC3.2.1.21) were highly represented. A total of 113 genes in the DS-ZC vs. CK-ZC group and 121 genes in the DS-ZY vs. CK-ZY group were identified as significantly changed in the starch and sucrose metabolism pathway, with the genes related to β-glucosidase (EC3.2.1.21) being the most represented. In the DS-ZC vs. CK-ZC and DS-ZY vs. CK-ZY groups, most of the β-glucosidase-related genes were significantly downregulated by drought stress. In the DS-ZC vs. CK-ZC group, of the genes related to β-glucosidase, 2 were downregulated and 1 upregulated in the DS7 and DS14 time points, 6 downregulated and 2 upregulated in the DS21 time point, 7 downregulated and 1 upregulated in the DS28 time point, and 9 downregulated and 3 upregulated in the DS35 time point, respectively. In the DS-ZY vs. CK-ZY group, of the genes related to β-glucosidase, 2 were downregulated and 1 upregulated in the DS7 time point, 3 downregulated in the DS14 time point, 10 downregulated in the DS21 time point, 13 downregulated and 2 upregulated in the DS28 time point, and 8 downregulated and 1 upregulated in the DS35 time point, respectively.

### 3.11 Identification of co-expressed and specifically expressed DEGs

**Figure 10:**
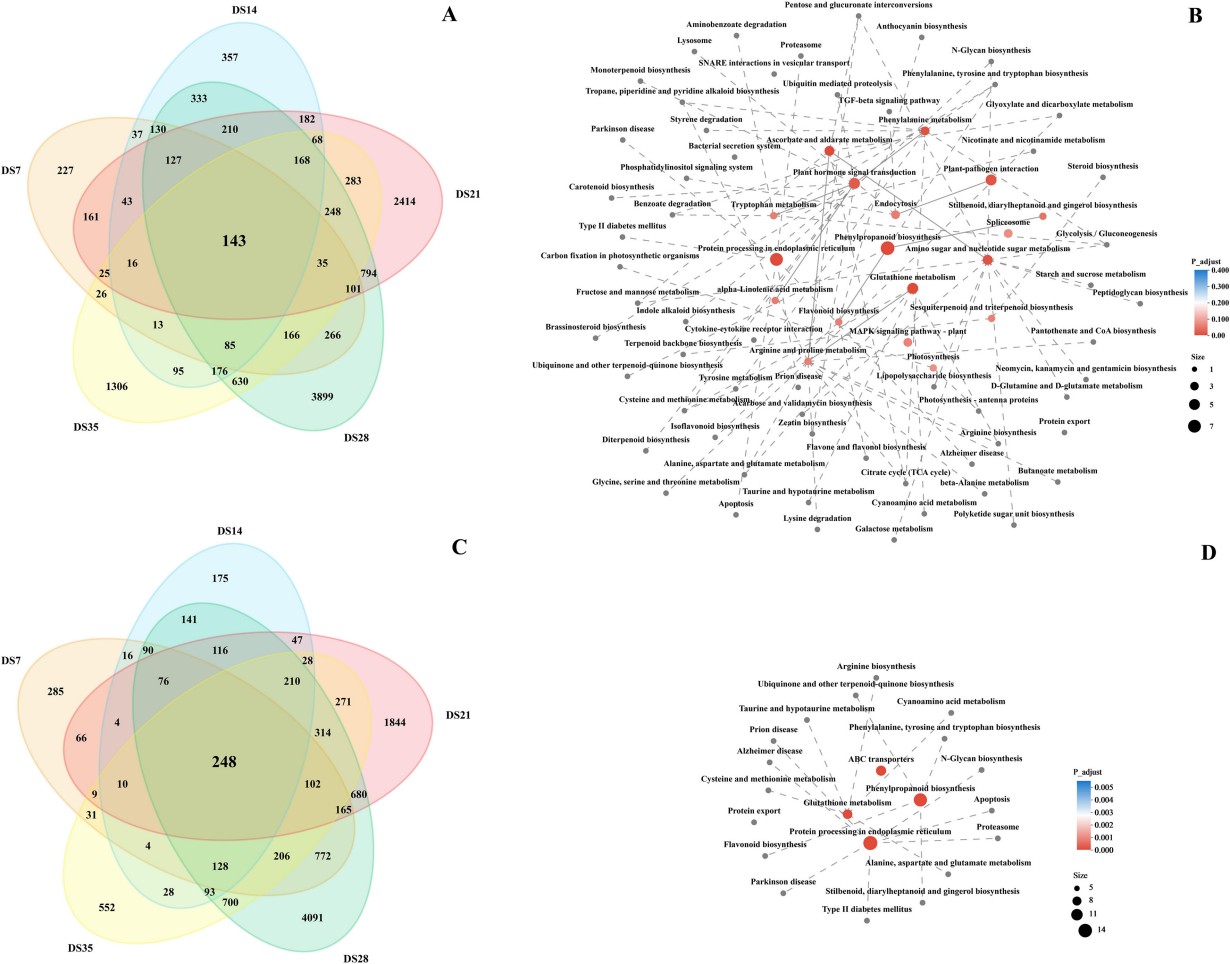
Venn diagram showing co-expressed or specifically expressed differentially expressed genes in DS-ZC vs CK-ZC **(A)** and DS-ZY vs CK-ZY **(C)** groups at different drought stress stages. The KEGG enrichment analysis networks of co-expressed differentially expressed genes in DS-ZC vs CK-ZC **(B)** and DS-ZY vs CK-ZY **(D)** groups. ZC, ZC208 genotype; ZY, ZY100 genotype; CK, well-watered; DS, drought stress. DS7, DS14, DS21, DS28 and DS35 indicate the plants suffered drought stress for 7, 14, 21, 28 and 35 days, respectively.

To identify co-expressed DEGs in the five drought stress time points, we constructed Venn diagrams for the DS-ZC vs. CK-ZC and the DS-ZY vs. CK-ZY group (**Figure 10**). We observed 221, 357, 2414, 3899, and 1306 unique DEGs in the DS7, DS14, DS21, DS28, and DS35 time points, respectively, and 143 common DEGs among the five time points in the DS-ZC vs. CK-ZC group (**Figure 10A**). For the DS-ZY vs. CK-ZY group, there were 285, 175, 1844, 4091, and 552 unique DEGs in the DS7, DS14, DS21, DS28, and DS35 time points, respectively, and 248 common DEGs among the five time points (**Figure 10C**). The 143 DEGs in the DS-ZC vs. CK-ZC group and 248 DEGs in the DS-ZY vs. CK-ZY group indicated substantial overlap in transcriptomic changes across the five drought time points.

**Figure 11:**
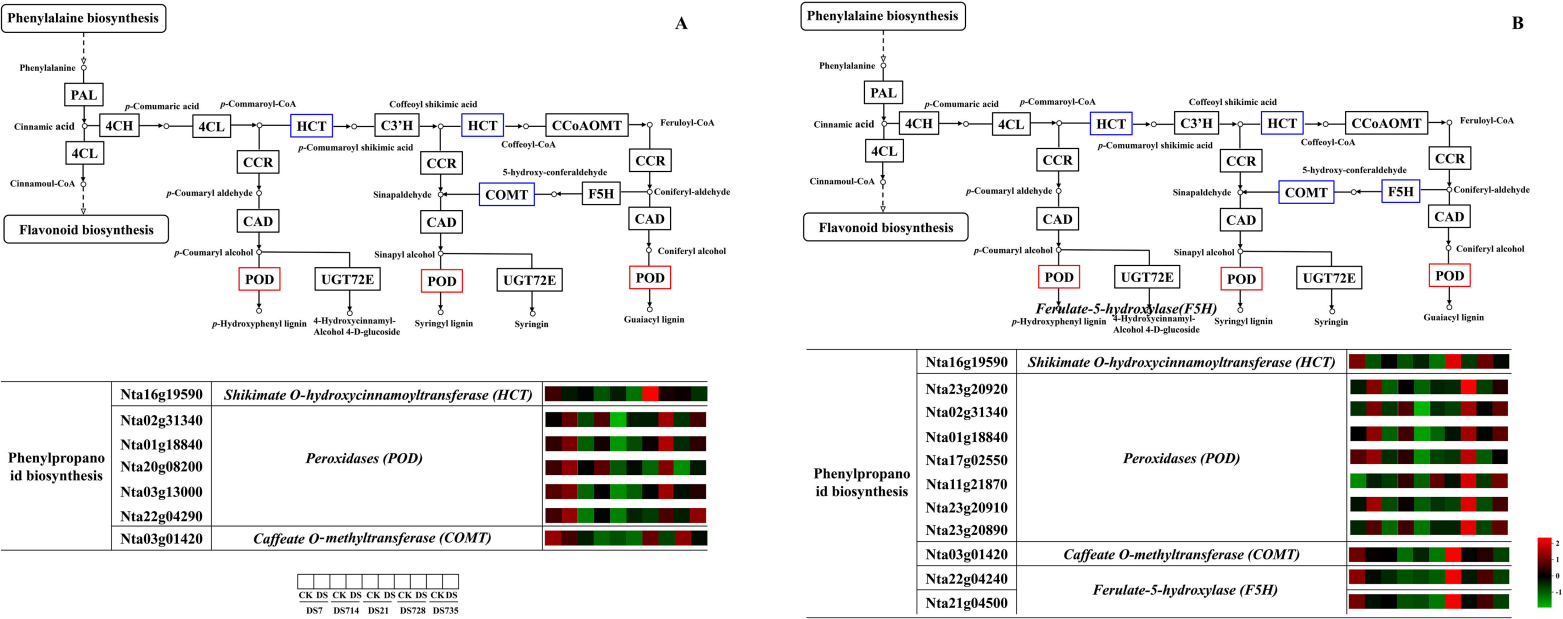
KEGG enriched differentially expressed genes in DS-ZC vs CK-ZC **(A)** and DS-ZY vs CK-ZY **(B)** groups involved in phenylpropanoid biosynthesis. The expression levels of genes are shown from green to red (low to high) in the comparisons of CS72 vs. CK, respectively. The scales are generated using log2(fold change) values. Genes marked with red and blue color in the border of matrix represent up-regulation and down-regulation of differentially expressed genes, respectively.

To further investigate the potential functions of the 143 and 248 DEGs, we constructed KEGG enrichment analysis networks. As illustrated in **Figure 10B**, the 143 common DEGs in the DS-ZC vs. CK-ZC group were predominantly enriched in pathways such as “phenylpropanoid biosynthesis”, “protein processing in the endoplasmic reticulum”, “plant hormone signal transduction”, “glutathione metabolism”, “plant-pathogen interaction”, “phenylalanine metabolism”, and “ascorbate and aldarate metabolism.” In the DS-ZY vs. CK-ZY group, the 248 common DEGs were primarily enriched in “phenylpropanoid biosynthesis”, “protein processing in the endoplasmic reticulum”, “glutathione metabolism”, and “ABC transporters” (**Figure 10D**). Interestingly, even though the number of common DEGs identified in the DS-ZC vs. CK-ZC group was significantly lower than that in the DS-ZY vs. CK-ZY group, it displayed a more intricate metabolic pathway network. Given the crucial role of phenylpropanoid biosynthesis in drought stress response, the relative abundance of DEGs associated with this pathway is presented in **Figure 11**. In the DS-ZC vs. CK-ZC group, the relative abundance of DEGs encoding shikimate O-hydroxycinnamoyltransferase (HCT) and caffeate O-methyltransferase (COMT) was downregulated, while PODs were upregulated. Similarly, in the DS-ZY vs. CK-ZY group, DEGs involved in the biosynthesis of phenylpropanoid, including HCT, COMT, and ferulate-5-hydroxylase (F5H), were downregulated, whereas POD expression increased due to drought stress.

### 3.12 Weighted gene coexpression network analysis

**Figure 12:**
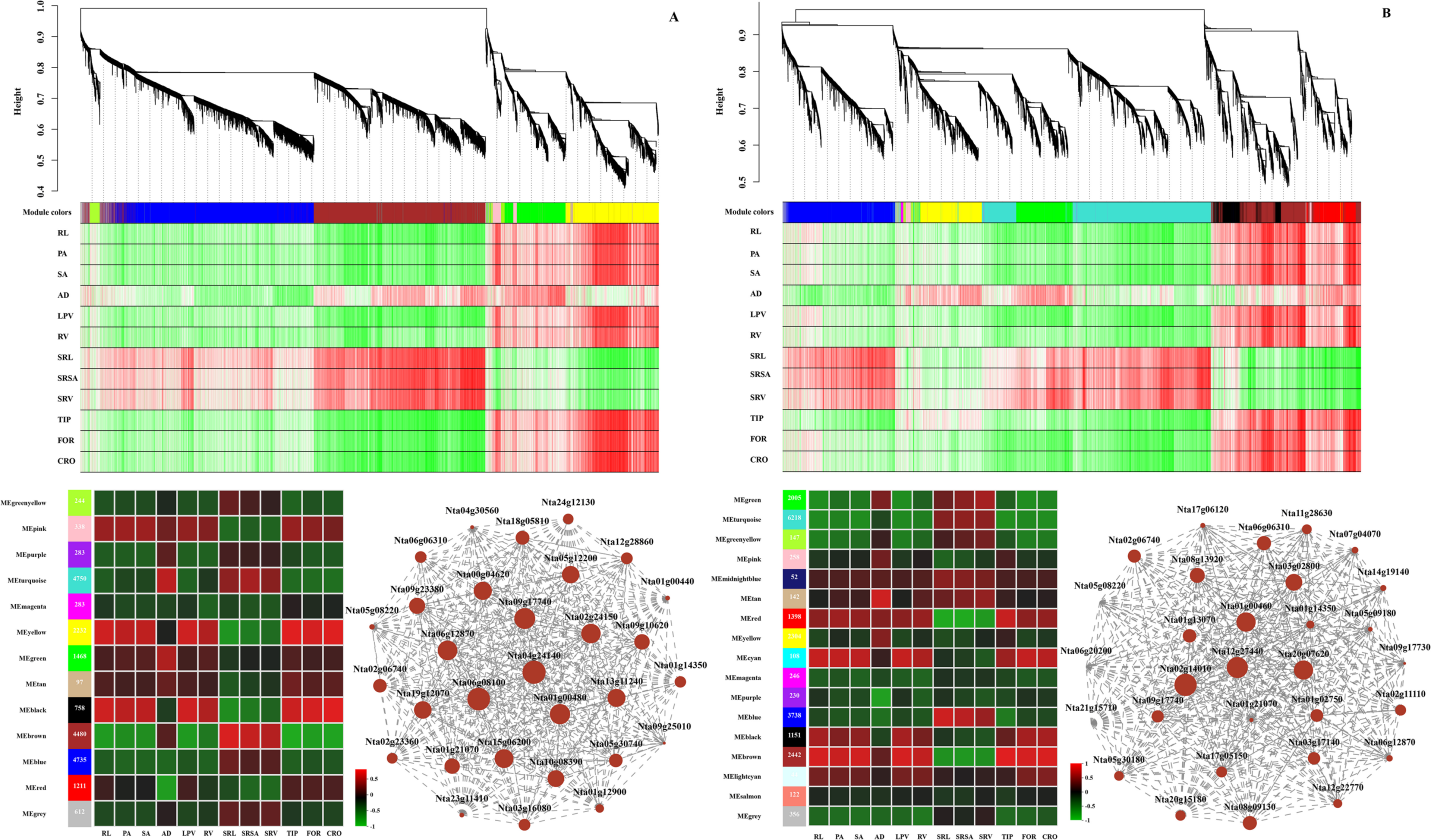
Weighted gene coexpression network analysis (WGCNA) of *Nicotiana tabacum* under different drought stress treatments. Gene clustering dendrogram and module assignment from WGCNA for ZC208 genotype **(A)** and ZY100 genotype **(B)**. For each WGCNA network, hierarchical cluster trees show different co-expression modules labelled by different colors based on the gene expression clustering results. The bottom heatmaps indicate the correlation analysis between identified modules and different physiological traits. Each row corresponds to a module and each column corresponds to a physiological index. The colors varied from blue to red represent the scale of correlation co-efficients, ranging from -1 (green) to 1 (red). The bottom co-occurrence networks present the relationship between top 30 hub genes.

To thoroughly investigate the transcriptional responses of *N. tabacum* under drought stress, we conducted a Weighted Gene Coexpression Network Analysis (WGCNA) to identify gene sets or functional pathways associated with the RSA. The dynamic tree cut results revealed 13 distinct modules containing 97 to 4735 genes in drought-stressed ZC208 (**Figure 12A**) and 17 distinct modules with 44 to 6218 genes in drought-stressed ZY100 (**Figure 12B**). Based on the correlation coefficients and abundance levels of characteristic genes from each module in relation to RSA parameters in ZC208, most parameters (excluding SRL, SRSA, and SRV) were strongly and positively correlated with the MEyellow module (2232 genes) and the MEblack module (758 genes). In the ZY100 cultivar, the MEbrown module (2442 genes) exhibited a strong positive correlation with most RSA parameters, including RL, PA, SA, AD, LPV, RV, TIP, FOR, and CRO, while showing a significantly negative correlation with SRL, SRSA, and SRV.

We then conducted a KEGG enrichment analysis on the genes within the MEyellow and MEblack modules in ZC208, as well as the MEbrown module in ZY100. As illustrated in **Supplementary Figure 5A**, the genes in the MEyellow and MEblack modules of ZC208 were significantly enriched in pathways such as ‘MAPK signaling pathway, plant’ (map04016), ‘plant-pathogen interaction’ (map04626), and ‘sesquiterpenoid and triterpenoid biosynthesis’ (map00909). In contrast, the genes in the MEbrown module of ZY100 were primarily enriched in ‘amino sugar and nucleotide sugar metabolism’ (map00520), ‘MAPK signaling pathway, plant’ (map04016), and ‘plant-pathogen interaction’ (map04626) (**Supplementary Figure 5B**). Given the strong correlations between the MEyellow and MEblack modules and RSA in ZC208, as well as the significant relationship between the MEbrown module and RSA in ZY100, we constructed gene networks to identify hub genes with the highest connectivity. The top genes exhibiting the greatest connectivity were selected as candidate hub genes for constructing the network. As shown in **Figure 12**, all identified genes were closely interconnected, with the hub gene *Nta04g24140* in ZC208 and *Nta02g14010* in ZY100 demonstrating the highest connectivity, respectively.

### 3.13 qRT-PCR validation

To validate the RNA-Seq data, qRT-PCR was performed on 15 randomly selected DEGs. A strong concordance was observed between the expression profiles obtained from qRT-PCR and RNA-Seq (**Supplementary Figure 6**). Specifically, a Pearson correlation analysis of the log2(fold change) values revealed high consistency, with coefficients (r) ranging from 0.79 to 0.99 for ZC208 and 0.60 to 0.96 for ZY100 (**Supplementary Figure 7**). These results collectively affirm the reliability of the transcriptomic dataset.

## 4 Discussion

Drought stress, a major abiotic factor exacerbated by global climate change, severely impacts plant growth, development, and agricultural productivity worldwide (Kapoor et al., 2020; Spinoni et al., 2018). Our study comprehensively investigated the time-course adaptation strategies of two *N. tabacum* varieties, drought-tolerant ZC208 and drought-sensitive ZY100, focusing on root system architecture, physiological responses, and transcriptomic regulations under varying drought conditions.

### 4.1 Morphological and physiological changes of *N. tabacum* roots under drought stress

Drought stress significantly impacted the morphological and physiological characteristics of *N. tabacum* roots, with notable differences observed between the tolerant (ZC208) and sensitive (ZY100) varieties. At the cellular level, prolonged drought led to severe disruption of root cell ultrastructure, characterized by vacuolization, organelle damage, and the accumulation of autolysosome-like structures and mucilage (Zhang et al., 2025). These changes are indicative of cellular stress and degradation, consistent with the adverse effects of water deficit on membrane integrity and tissue viability (Zhu, 2016). These results are consistent because drought stress universally causes cellular damage (like organelle degradation and membrane disruption) due to dehydration and oxidative stress. The observed ultrastructural changes (vacuolization, autophagic structures) are typical stress responses across plant species when water deficit disrupts cellular homeostasis.

Physiologically, drought stress induced a significant increase in oxidative damage, as evidenced by elevated levels of ROS, including O_2_^•^-^^, H_2_O_2_, and ^•^OH, and the lipid peroxidation product MDA (Cruz de Carvalho, 2008; Sato et al., 2024). The drought-sensitive ZY100 consistently exhibited higher levels of these oxidative stress markers compared to ZC208 (**Figure 2**), suggesting a reduced capacity to mitigate ROS-induced damage or a more pronounced stress response (Ren et al., 2024). This aligns with the understanding that ROS overproduction is a major consequence of abiotic stress, leading to cellular dysfunction (Miller et al., 2010). Interestingly, even under well-watered (CK) conditions, the sensitive variety ZY100 exhibited a gradual, time-dependent increase in O_2_^•^-^^, H_2_O_2_, ^•^OH, and MDA levels that was not observed in ZC208. This suggests that ZY100 may possess a less efficient constitutive antioxidant system or a higher baseline level of metabolic turnover, making it more susceptible to the accumulation of oxidative byproducts during normal development. Under drought stress, a striking contrast in regulation emerged: while ZY100 showed a continuous and progressive increase in ^•^OH and MDA, ZC208 demonstrated an ability to stabilize or ‘plateau’ these levels over time. Specifically, the relatively stable MDA levels in ZC208 suggest superior membrane protection and lipid peroxidation control, likely supported by its more synchronized activation of antioxidant enzymes (**Table 2**).

RB and RA were significantly reduced under drought conditions in both varieties, with ZY100 showing a greater decline than ZC208 (**Figure 3**). This reduction reflects a common plant strategy to conserve energy and water resources by limiting growth under stress (Gul et al., 2023; Nadeem et al., 2019). However, the dynamic changes in RSA represent a crucial adaptive response. While overall RSA parameters such as RL, PA, SA, AD, LPV, RV, TIP, FOR, and CRO generally decreased, while SRL, SRSA, and SRV significantly increased (**Table 1**). This shift towards a finer, more branched root system is a well-documented strategy for enhancing water and nutrient acquisition efficiency from deeper soil layers in arid environments (Gupta et al., 2021; Lynch, 2019; Shoaib et al., 2022). The observed differences in RSA adjustments between ZC208 and ZY100 suggest that the drought-tolerant variety might possess a more effective root plasticity to optimize water uptake. These findings reflect that reduced biomass allocation (RB/RA decline) as a common energy conservation response under stress, and increased root fineness (higher SRL, SRSA, SRV) as a classic morphological adaptation to enhance water foraging efficiency in drying soils. The variety-specific differences in root plasticity also align with reports that tolerant genotypes often exhibit more flexible root system remodeling to optimize resource capture.

To counter the physiological impacts of drought, roots activated robust defense mechanisms. Antioxidant enzyme activities, including SOD, POD, and CAT, significantly increased with drought duration, reaching their highest levels at the 35-day mark (**Table 2**). These enzymes are essential for scavenging ROS and maintaining cellular redox homeostasis (Cvetkovic et al., 2017; Miller et al., 2010; Pomeroy et al., 1975). Concurrently, proline content in roots was significantly enhanced, serving as a key osmolyte to maintain cell turgor, stabilize proteins and membranes, and scavenge ROS (Bashir et al., 2021; Florencio-Ortiz et al., 2018; Kaur and Asthir, 2015). Conversely, SS content was remarkably reduced, which could indicate their utilization as an energy source or conversion into other stress-related compounds, or a disruption in carbohydrate metabolism under severe stress (Cuellar-Ortiz et al., 2008). The observed increase in Pro and decrease in SS in drought-stressed *N. tabacum* roots (**Table 2**) suggest active proline accumulation for osmotic adjustment and ROS scavenging, while soluble sugars are likely consumed or their synthesis is impaired under these conditions.

### 4.2 Transcriptomic responses to drought stress

Transcriptomic analysis provided a molecular blueprint of the root’s response to drought stress, revealing a complex network of gene expression changes over time. A significant number of DEGs were identified, with a consistent trend of more downregulated than upregulated genes across all drought stages in both varieties (**Figure 4**). This pattern often reflects a plant’s strategy to suppress growth-related processes and reallocate resources towards stress adaptation and survival under adverse conditions (Skirycz and Inzé, 2010). GO enrichment analysis highlighted several key biological processes. Terms such as “sesquiterpene biosynthetic process,” “response to hydrogen peroxide,” and “protein self-association” were significantly enriched (**Figure 5**). Sesquiterpenoids are known secondary metabolites involved in plant defense responses (Yadav et al., 2021), while the “response to hydrogen peroxide” directly points to the activation of ROS scavenging mechanisms. “Protein self-association” and terms like “structural constituent of chromatin” and “nucleosome” suggest active processes of protein stabilization, cellular repair, and chromatin remodeling, which are crucial for maintaining cellular integrity and regulating gene expression under stress (Zafar et al., 2024). KEGG pathway enrichment analysis further elucidated the metabolic and signaling pathways involved. Pathways such as ‘sesquiterpenoid and triterpenoid biosynthesis’, ‘phenylpropanoid biosynthesis’, ‘taurine and hypotaurine metabolism’, ‘DNA replication’, ‘starch and sucrose metabolism’, and ‘glutathione metabolism’ were significantly enriched (**Figure 6**). Phenylpropanoid biosynthesis is a critical pathway for the synthesis of lignin and flavonoids, which contribute to cell wall strengthening, reduced water loss, and antioxidant defense (Shao et al., 2023) (Wang et al., 2024). The involvement of “starch and sucrose metabolism” pathways, despite the observed reduction in soluble sugars, indicates a dynamic regulation of carbohydrate partitioning and utilization to provide energy and osmolytes for stress adaptation (Hu et al., 2021). “Glutathione metabolism” is directly linked to the plant’s antioxidant defense system, playing a vital role in detoxifying ROS (Sato et al., 2024).

Time-course transcriptomic analysis using Short Time-series Expression Miner (STEM) identified distinct gene expression profiles for both ZC208 (6 profiles) and ZY100 (8 profiles) (**Figure 7**), demonstrating the dynamic nature of gene regulation during drought progression. Notably, downregulated profiles (e.g., profile 0) and upregulated profiles (e.g., profile 19) were enriched in common pathways such as ‘phenylpropanoid biosynthesis’, ‘starch and sucrose metabolism’, ‘plant hormone signal transduction’, and ‘protein processing in the endoplasmic reticulum’ (**Figure 7**). This temporal regulation of gene expression is crucial for plants to fine-tune their responses as stress intensity and duration change. Differential expression patterns of genes related to plant hormone signaling (e.g., AUX/IAA, PYR/PYL) and various enzymes in starch and sucrose metabolism (e.g., endoglucanase, β-glucosidase) further underscore the central regulatory roles of hormones and carbohydrate metabolism in drought adaptation. This temporal regulation of gene expression aligns with previous studies demonstrating that plants dynamically reprogram transcription as drought progresses, enabling phased activation of defense pathways (Li et al., 2021). The enrichment of both up- and down-regulated gene sets in common pathways (e.g., phenylpropanoid biosynthesis, hormone signaling) is consistent with reports that these pathways undergo complex, time-dependent regulation rather than simple linear activation under stress (Ullah et al., 2018). The involvement of hormone signaling genes (AUX/IAA, PYR/PYL) and carbohydrate metabolism enzymes further corroborates established models positioning phytohormones and energy metabolism as central hubs coordinating drought adaptation (Wang et al., 2021).

### 4.3 Correlation analysis between RSA traits and DEGs in drought stressed

The integration of transcriptomic data with physiological and morphological traits through Weighted Gene Co-expression Network Analysis (WGCNA) provided a systems-level understanding of drought adaptation. WGCNA identified specific gene modules (e.g., MEyellow and MEblack in ZC208; MEbrown in ZY100) that were strongly correlated with RSA parameters (**Figure 12**). These modules were significantly enriched in pathways such as ‘MAPK signaling pathway, plant’, ‘plant-pathogen interaction’, and ‘sesquiterpenoid and triterpenoid biosynthesis’. The MAPK signaling pathway is a well-established cascade involved in mediating plant responses to various abiotic stresses, including drought, by transducing external signals into cellular responses. The strong correlations between these gene networks and RSA parameters suggest that the observed morphological adjustments in root architecture are not merely passive responses but are actively regulated by complex molecular pathways, enabling plants to optimize water acquisition under water-limited conditions (Siddiqui et al., 2020). This WGCNA-based analysis is consistent with previous studies by confirming MAPK signaling and secondary metabolism as key drought-responsive pathways (Wang et al., 2023), and root architectural changes are actively regulated by gene networks, not passive (Lynch, 2013), demonstrating genotype-specific module-trait correlations, reflecting known diversity in stress adaptation mechanisms.

Further analysis of common DEGs across all drought time points revealed predominant enrichment in ‘phenylpropanoid biosynthesis’, ‘protein processing in the endoplasmic reticulum’, ‘plant hormone signal transduction’, and ‘glutathione metabolism’ (**Figure 10**). Although the number of common DEGs was lower in ZC208 than in ZY100, the metabolic pathway network in ZC208 appeared more intricate, potentially indicating a more refined and diverse regulatory mechanism in the drought-tolerant variety. The differential regulation of genes involved in phenylpropanoid biosynthesis, such as the downregulation of shikimate O-hydroxycinnamoyltransferase (HCT), caffeate O-methyltransferase (COMT), and ferulate-5-hydroxylase (F5H), coupled with the upregulation of POD, suggests a strategic adjustment in secondary metabolite synthesis to enhance drought resistance (Wang et al., 2024). This intricate interplay between gene expression, metabolic pathways, and root morphological changes highlights the sophisticated adaptive strategies employed by *N. tabacum* roots to cope with drought stress.

## 5 Conclusions

This study provides a comprehensive time-course analysis of the physiological, morphological, and molecular responses of *N. tabacum* roots to drought stress, highlighting distinct adaptive strategies in drought-tolerant (ZC208) and drought-sensitive (ZY100) varieties. Drought stress severely impaired root cell ultrastructure, increased oxidative damage, and reduced root biomass and activity, with the sensitive variety showing greater vulnerability. Plants adapted by modifying root system architecture, enhancing antioxidant enzyme activities (SOD, POD, CAT), and accumulating osmolytes like proline. Transcriptomic analyses, including time-course (STEM) and co-expression network (WGCNA) approaches, revealed complex gene regulatory networks and key pathways that underpin drought tolerance. These included ‘sesquiterpenoid and triterpenoid biosynthesis’, ‘phenylpropanoid biosynthesis’, ‘response to hydrogen peroxide’, ‘plant hormone signal transduction’, ‘starch and sucrose metabolism’, and ‘glutathione metabolism’. The strong correlations between specific gene modules and root system architecture parameters underscore the molecular control over root morphological plasticity, which is crucial for optimizing water acquisition. These findings offer valuable insights into the intricate mechanisms underlying drought tolerance in tobacco roots. The identified genes and pathways serve as potential targets for future biotechnological interventions and breeding programs aimed at developing drought-resilient tobacco cultivars, thereby contributing to agricultural sustainability in a changing climate.

## Data Availability

The data presented in the study are deposited in the NCBI BioProject repository, accession number PRJNA1443673.
