# Supplementary figures and images for: Time-course transcriptomic information unravels the adaptation strategies of Nicotiana tabacum to drought stress through altered root system architecture
Source: Front Plant Sci. 2026 Apr 6;17:1781718. doi: 10.3389/fpls.2026.1781718 (PMC13094138; doi:10.3389/fpls.2026.1781718)

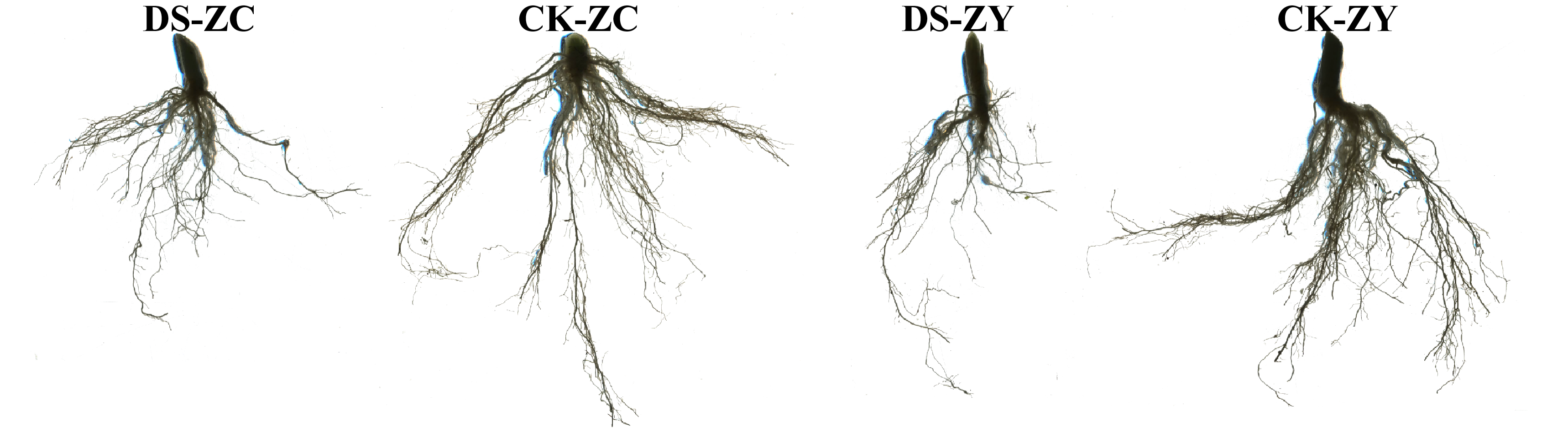

Supplement: Supplementary file 1 [file Image1.png]

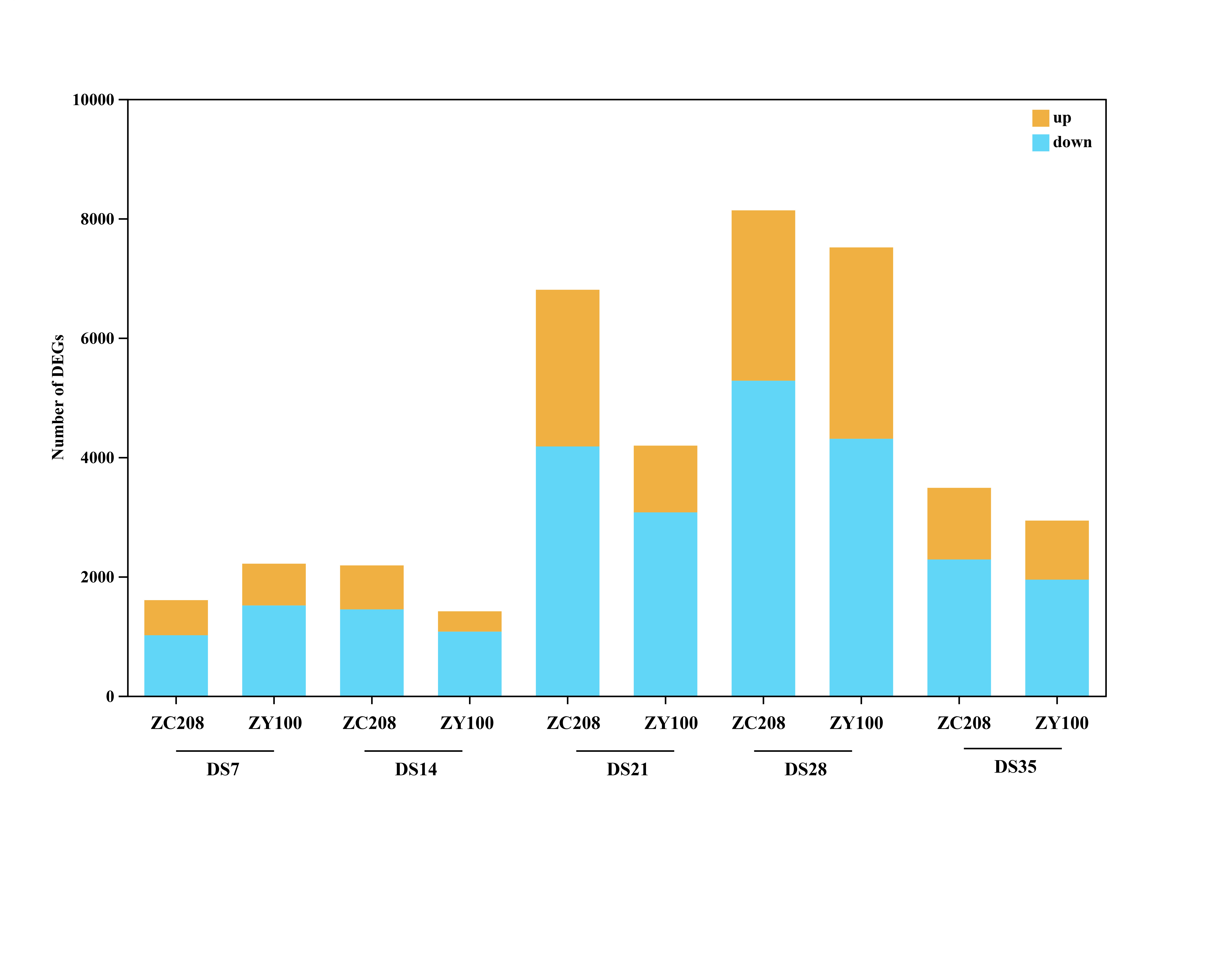

Supplement: Supplementary file 4 [file Image4.jpeg]

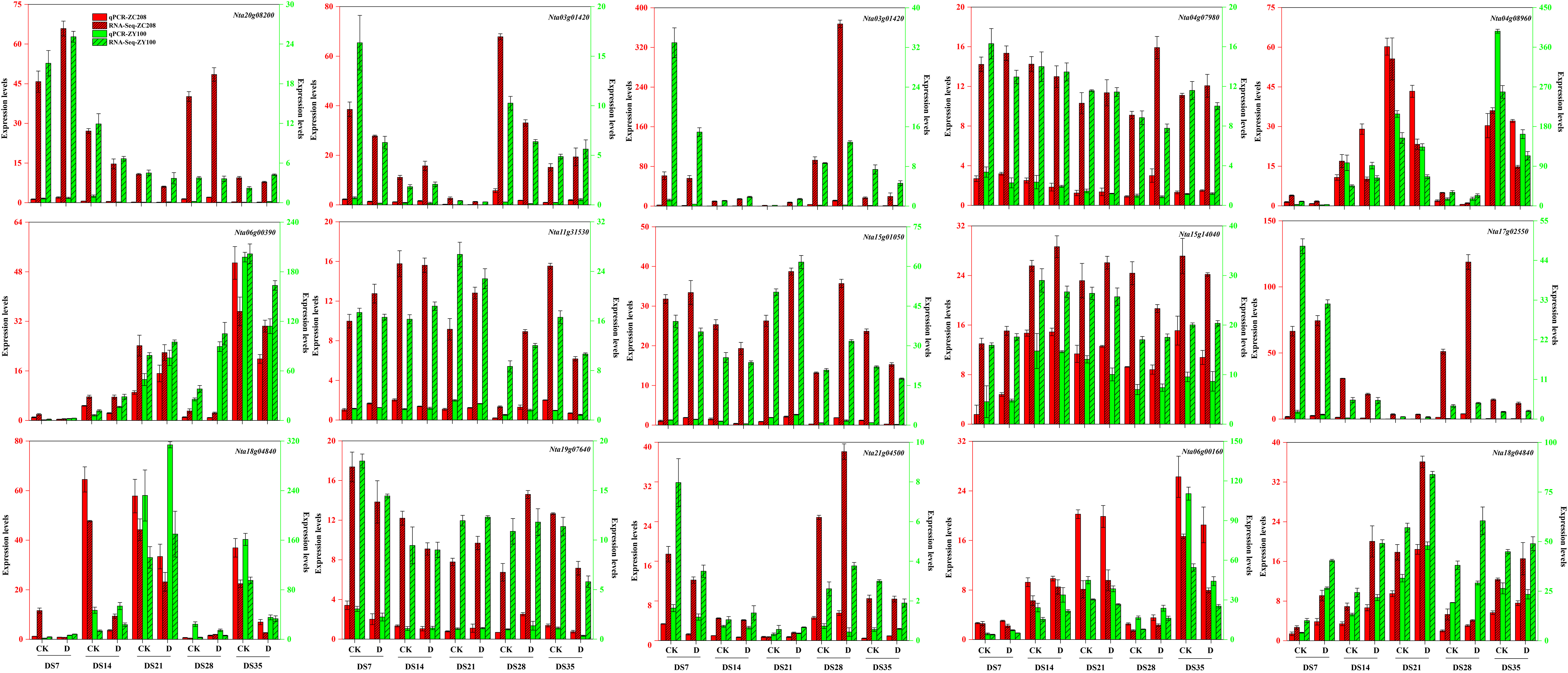

Supplement: Supplementary file 6 [file Image6.jpeg]

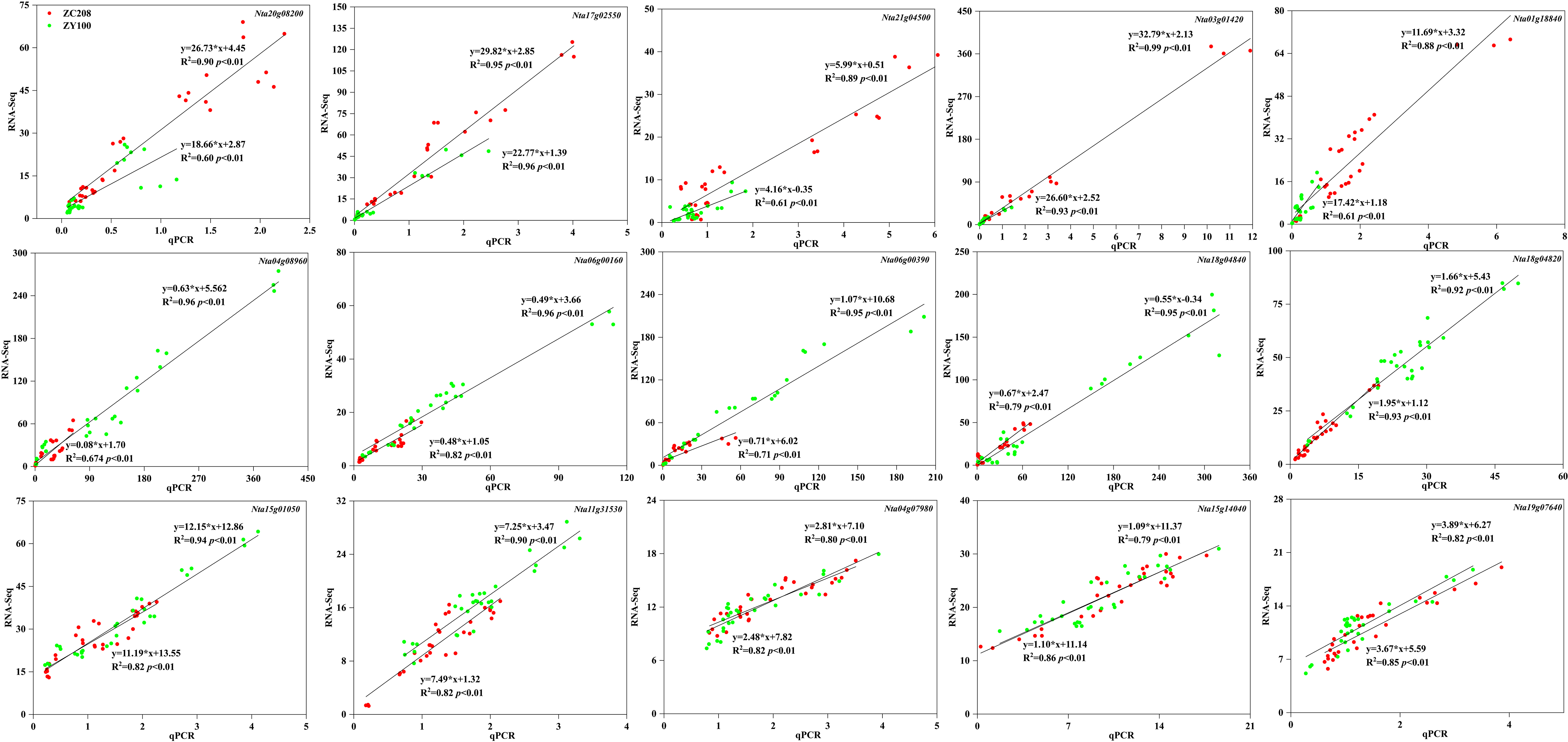

Supplement: Supplementary file 7 [file Image7.jpeg]
