# Supplementary material for: Time-course transcriptomic information unravels the adaptation strategies of Nicotiana tabacum to drought stress through altered root system architecture
Source: Front Plant Sci. 2026 Apr 6;17:1781718. doi: 10.3389/fpls.2026.1781718 (PMC13094138; doi:10.3389/fpls.2026.1781718)
Supplement: Supplementary file 8 [file Table1.docx]

**Table S1 The basic physical and chemical properties of surface soil.**

| Soil texture | Sand (%) | 72.1 |
| --- | --- | --- |
|  | Silt (%) | 13.2 |
|  | Clay (%) | 14.9 |
| pH value | 6.78 | |
| Organic matter content (g kg^-1^) | 14.53 | |
| Total nitrogen content (mg kg^-1^) | 78.24 | |
| Available phosphorus content (mg kg^-1^) | 82.36 | |
| Available potassium content (mg kg^-1^) | 184.66 | |

**Table S2 qRT-PCR primer sequence of differentially expressed genes in transgenic alfalfa under salt stress.**

| **Gene name** | **Description** | **Forward primer (5′-3′)** | **Reverse primer (3′-5′)** |
| --- | --- | --- | --- |
| Nta20g08200 | cationic peroxidase 1-like | AGCACAGCCAACAATGACATTC | ACGAGGTCCTTCTCATCCAAAC |
| Nta17g02550 | peroxidase 21-like | ACGTGTCGAAATGAGAACAGGA | ATAAAACTGCCTCTGGGTCAGG |
| Nta21g04500 | cytochrome P450 84A1-like | AAGATCTTGCCCAGGTATGCAA | TCATTCCATCAGGCAATTCCCA |
| Nta03g01420 | caffeic acid 3-O-methyltransferase-like | CTGGTGTTGAGCATGTTTCAGG | CGTTCTTTCTTTCCCTCCAGGA |
| Nta01g18840 | peroxidase N1 | TTCTTCCTCAGCTTCGAGCATT | TCCACAGCTTCTGGTCTGATTC |
| Nta04g08960 | 18.2 kDa class I heat shock protein-like | TTTGAAGGTTTCCCCCTTTCCA | TGCCACTGCTCATTCTTCTCTT |
| Nta06g00160 | stromal 70 kDa heat shock-related protein | TCAATGACTCCCAGAGAACTGC | AATTTTCAGCAAGCCAGTCGAC |
| Nta06g00390 | 22.0 kDa class IV heat shock protein-like | AAGCTTGAGAATGGGGTGCTAA | TCTTCTTTGGTCTCAATGGCGA |
| Nta15g19530 | 17.3 kDa class II heat shock protein-like | GACGTGAAAGAGTACCCCAACT | ATTGTCATCTTCCACCTGCACT |
| Nta06g13600 | heat shock protein 82-like | GCAGCTCATCAACAAGCAGAAA | CTCTCTTGGGCACAAAGAGGAT |
| Nta18g04840 | 17.6 kDa class I heat shock protein-like | ATTGGAAGGAAACGCAAGAAGC | ACCCCATTTTCCATACAAGCCT |
| Nta18g04820 | 18.1Kda class I heat shock protein-like | ACGCAGCAGTGGAAAATTCTTC | TTCAACATCTCTGAGGTGGTGG |
| Nta15g01050 | auxin response factor 9-like isoform X1 | ATTCACACTCAGCTCCTGGC | GCTCGTATCCGATGCAGTCA |
| Nta11g31530 | two-component response regulator ORR24-like | AGCAGAGTAAACGTGCCATACA | ATTTCCAAATCCTCCCCCTGTC |
| Nta04g07980 | BES1/BZR1 homolog protein 2-like | CATCATCCTCATTCCCTAGCCC | GAGGAAAGAGGTGGGGTAACAG |
