# Supplementary material for: Time-course transcriptomic information unravels the adaptation strategies of Nicotiana tabacum to drought stress through altered root system architecture
Source: Front Plant Sci. 2026 Apr 6;17:1781718. doi: 10.3389/fpls.2026.1781718 (PMC13094138; doi:10.3389/fpls.2026.1781718)
Supplement: Supplementary file 10 [file Table3.docx]

**Table S3.** Summary statistics of quality control and mapping from sequencing of *Nicotiana tabacum*.

| Drought stress days | Sample | Raw reads | Raw bases | Clean reads | Clean bases | Error rate(%) | Q20(%) | Q30(%) | GC content(%) |
| --- | --- | --- | --- | --- | --- | --- | --- | --- | --- |
| DS7 | CK-ZC208-1 | 46588516 | 7.03E+09 | 46201292 | 6.86E+09 | 0.0116 | 99.03 | 96.82 | 42.49 |
|  | CK-ZC208-2 | 49325784 | 7.45E+09 | 48901852 | 7.3E+09 | 0.0116 | 99.01 | 96.79 | 42.48 |
|  | CK-ZC208-3 | 45070050 | 6.81E+09 | 44776396 | 6.67E+09 | 0.0115 | 99.04 | 96.85 | 42.32 |
|  | D-ZC208-1 | 42928370 | 6.48E+09 | 42577800 | 6.35E+09 | 0.0116 | 99.01 | 96.76 | 42.41 |
|  | D-ZC208-2 | 48927438 | 7.39E+09 | 48608946 | 7.24E+09 | 0.0116 | 99.03 | 96.83 | 42.25 |
|  | D-ZC208-3 | 44040206 | 6.65E+09 | 43663520 | 6.51E+09 | 0.0116 | 99.02 | 96.82 | 42.35 |
|  | CK-ZY100-1 | 54450302 | 8.22E+09 | 54031488 | 8.03E+09 | 0.0116 | 99 | 96.75 | 42.39 |
|  | CK-ZY100-2 | 49746460 | 7.51E+09 | 49364812 | 7.37E+09 | 0.0116 | 98.98 | 96.67 | 42.52 |
|  | CK-ZY100-3 | 51295746 | 7.75E+09 | 50773806 | 7.55E+09 | 0.0116 | 99.01 | 96.79 | 42.45 |
|  | D-ZY100-1 | 52588022 | 7.94E+09 | 52101740 | 7.74E+09 | 0.0116 | 99.01 | 96.79 | 42.43 |
|  | D-ZY100-2 | 50888914 | 7.68E+09 | 50447950 | 7.51E+09 | 0.0116 | 99.02 | 96.83 | 42.31 |
|  | D-ZY100-3 | 39809706 | 6.01E+09 | 39458046 | 5.88E+09 | 0.0116 | 99.02 | 96.81 | 42.34 |
|  |  |  |  |  |  |  |  |  |  |
| DS14 | CK-ZC208-1 | 53287662 | 8.05E+09 | 52855642 | 7.87E+09 | 0.0116 | 99.03 | 96.8 | 42.19 |
|  | CK-ZC208-2 | 44661282 | 6.74E+09 | 44303940 | 6.59E+09 | 0.0116 | 99 | 96.72 | 42.16 |
|  | CK-ZC208-3 | 47504332 | 7.17E+09 | 47082712 | 7.01E+09 | 0.0116 | 99 | 96.74 | 42.14 |
|  | D-ZC208-1 | 58308796 | 8.8E+09 | 57845938 | 8.6E+09 | 0.0115 | 99.04 | 96.87 | 42.26 |
|  | D-ZC208-2 | 48108270 | 7.26E+09 | 47711512 | 7.1E+09 | 0.0116 | 99.03 | 96.83 | 42.29 |
|  | D-ZC208-3 | 50746528 | 7.66E+09 | 50262572 | 7.47E+09 | 0.0116 | 99 | 96.75 | 42.29 |
|  | CK-ZY100-1 | 53847784 | 8.13E+09 | 53453430 | 7.95E+09 | 0.0115 | 99.04 | 96.85 | 42.19 |
|  | CK-ZY100-2 | 43147180 | 6.52E+09 | 42847660 | 6.44E+09 | 0.0116 | 99.01 | 96.78 | 42.37 |
|  | CK-ZY100-3 | 55471008 | 8.38E+09 | 55059736 | 8.18E+09 | 0.0116 | 99.02 | 96.79 | 42.29 |
|  | D-ZY100-1 | 52671034 | 7.95E+09 | 52254456 | 7.77E+09 | 0.0116 | 99.02 | 96.81 | 42.22 |
|  | D-ZY100-2 | 48903830 | 7.38E+09 | 48519644 | 7.23E+09 | 0.0116 | 98.99 | 96.71 | 42.26 |
|  | D-ZY100-3 | 51596004 | 7.79E+09 | 51204932 | 7.61E+09 | 0.0116 | 99.01 | 96.8 | 42.23 |
|  |  |  |  |  |  |  |  |  |  |
| DS21 | CK-ZC208-1 | 49159902 | 7.42E+09 | 48733752 | 7.26E+09 | 0.0116 | 99 | 96.77 | 42.31 |
|  | CK-ZC208-2 | 47254226 | 7.14E+09 | 46875506 | 6.99E+09 | 0.0116 | 99.02 | 96.78 | 42.13 |
|  | CK-ZC208-3 | 50118132 | 7.57E+09 | 49764054 | 7.43E+09 | 0.0116 | 99.02 | 96.82 | 42.11 |
|  | D-ZC208-1 | 47598256 | 7.19E+09 | 47209072 | 7E+09 | 0.0116 | 99.02 | 96.82 | 42.19 |
|  | D-ZC208-2 | 50220108 | 7.58E+09 | 49832590 | 7.4E+09 | 0.0116 | 99.02 | 96.78 | 42.09 |
|  | D-ZC208-3 | 50850272 | 7.68E+09 | 50440298 | 7.51E+09 | 0.0116 | 99.02 | 96.8 | 42.21 |
|  | CK-ZY100-1 | 48342202 | 7.3E+09 | 47942642 | 7.14E+09 | 0.0116 | 99.01 | 96.78 | 42.14 |
|  | CK-ZY100-2 | 50312216 | 7.6E+09 | 49851648 | 7.43E+09 | 0.0116 | 99.02 | 96.8 | 42.26 |
|  | CK-ZY100-3 | 49325004 | 7.45E+09 | 48902418 | 7.28E+09 | 0.0116 | 99.02 | 96.81 | 42.19 |
|  | D-ZY100-1 | 52288614 | 7.9E+09 | 51827620 | 7.69E+09 | 0.0116 | 99.02 | 96.8 | 42.16 |
|  | D-ZY100-2 | 43965462 | 6.64E+09 | 43593120 | 6.52E+09 | 0.0116 | 99.02 | 96.8 | 42.15 |
|  | D-ZY100-3 | 44186566 | 6.67E+09 | 43830184 | 6.54E+09 | 0.0116 | 99.03 | 96.81 | 42.15 |
|  |  |  |  |  |  |  |  |  |  |
| DS28 | CK-ZC208-1 | 52124026 | 7.87E+09 | 51698472 | 7.68E+09 | 0.0116 | 98.99 | 96.69 | 42.18 |
|  | CK-ZC208-2 | 49648758 | 7.5E+09 | 49315458 | 7.37E+09 | 0.0115 | 99.04 | 96.87 | 42.12 |
|  | CK-ZC208-3 | 50194764 | 7.58E+09 | 49806174 | 7.41E+09 | 0.0116 | 99.02 | 96.82 | 42.27 |
|  | D-ZC208-1 | 43141542 | 6.51E+09 | 42741850 | 6.38E+09 | 0.0116 | 99.01 | 96.77 | 42.26 |
|  | D-ZC208-2 | 55625308 | 8.4E+09 | 55170358 | 8.19E+09 | 0.0116 | 99.02 | 96.8 | 41.99 |
|  | D-ZC208-3 | 48225706 | 7.28E+09 | 47795940 | 7.11E+09 | 0.0116 | 99.02 | 96.78 | 42.17 |
|  | CK-ZY100-1 | 45452864 | 6.86E+09 | 45115804 | 6.71E+09 | 0.0116 | 99 | 96.72 | 42.25 |
|  | CK-ZY100-2 | 55644586 | 8.4E+09 | 55128366 | 8.2E+09 | 0.0116 | 99.02 | 96.81 | 42.24 |
|  | CK-ZY100-3 | 51801084 | 7.82E+09 | 51413068 | 7.65E+09 | 0.0116 | 99.02 | 96.82 | 42.22 |
|  | D-ZY100-1 | 53919224 | 8.14E+09 | 53379084 | 7.97E+09 | 0.0116 | 99.01 | 96.77 | 42.23 |
|  | D-ZY100-2 | 58913520 | 8.9E+09 | 58396048 | 8.68E+09 | 0.0115 | 99.03 | 96.85 | 42.24 |
|  | D-ZY100-3 | 53464850 | 8.07E+09 | 52847788 | 7.87E+09 | 0.0116 | 99.01 | 96.78 | 42.25 |
|  |  |  |  |  |  |  |  |  |  |
| DS35 | CK-ZC208-1 | 49734030 | 7.51E+09 | 49274554 | 7.34E+09 | 0.0116 | 99 | 96.72 | 41.82 |
|  | CK-ZC208-2 | 47790972 | 7.22E+09 | 47382656 | 7.07E+09 | 0.0116 | 99.02 | 96.81 | 41.57 |
|  | CK-ZC208-3 | 45554268 | 6.88E+09 | 45122004 | 6.71E+09 | 0.0116 | 99.01 | 96.76 | 41.85 |
|  | D-ZC208-1 | 44518662 | 6.72E+09 | 44144308 | 6.58E+09 | 0.0116 | 99.02 | 96.81 | 41.57 |
|  | D-ZC208-2 | 48020680 | 7.25E+09 | 47653390 | 7.09E+09 | 0.0116 | 99.02 | 96.8 | 41.53 |
|  | D-ZC208-3 | 49233210 | 7.43E+09 | 48860976 | 7.27E+09 | 0.0116 | 99.02 | 96.82 | 41.69 |
|  | CK-ZY100-1 | 43803620 | 6.61E+09 | 43436016 | 6.46E+09 | 0.0116 | 99.01 | 96.77 | 42.13 |
|  | CK-ZY100-2 | 47754402 | 7.21E+09 | 47365612 | 7.03E+09 | 0.0116 | 99 | 96.74 | 41.98 |
|  | CK-ZY100-3 | 54178556 | 8.18E+09 | 53736694 | 8.01E+09 | 0.0116 | 99.01 | 96.77 | 41.94 |
|  | D-ZY100-1 | 47782448 | 7.22E+09 | 47409910 | 7.06E+09 | 0.0116 | 99.01 | 96.76 | 42.07 |
|  | D-ZY100-2 | 45134158 | 6.82E+09 | 44844394 | 6.66E+09 | 0.0115 | 99.04 | 96.88 | 42 |
|  | D-ZY100-3 | 45623990 | 6.89E+09 | 45216574 | 6.75E+09 | 0.0116 | 98.99 | 96.7 | 42.11 |
